# The general and specific in anxiety: an ERP study of social and spatial anxiety

**DOI:** 10.3389/fnhum.2026.1837154

**Published:** 2026-07-09

**Authors:** Evgeniia Alenina, Kristina Terenteva, Vladimir Kosonogov

**Affiliations:** 1 Affective Psychophysiology Laboratory, Institute of Health Psychology, HSE University, Saint Petersburg, Russia; 2 Faculty of Biology and Biotechnology, HSE University, Moscow, Russia

**Keywords:** anxiety, EEG, ERP, social anxiety, spatial anxiety

## Abstract

Anxiety, characterized by pervasive feelings of nervousness and worry, may differentially impact cognitive problem-solving across various domains, including spatial and social contexts. This study investigates the relationship between distinct types of anxiety and cognitive performance, aiming to elucidate the underlying neural mechanisms involved. Sixty participants (54 females, Mage = 22.46, SD = 4.50; 6 males, Mage = 20.83, SD = 2.31) were divided into three groups: Control (*N* = 20), Social Anxiety (*N* = 20), and Spatial Anxiety (*N* = 20). During a one-hour electroencephalography (EEG) session, participants completed the Colour Stroop task, Flanker Spatial task, and Social Stroop task. Event-related potential (ERP) analysis revealed that the control group exhibited significantly lower amplitudes in N2, N3, early P3, LPP across all three tasks compared to both anxiety groups. No evidence was found for domain-specific effects. These findings suggest a shared neural processing mechanism of anxiety which encompasses different cognitive domains and highlights the complexity of interaction between different types of anxiety and cognitive performance.

## 1 Introduction

Anxiety is characterized by a complex interplay of worry, insecurity, and fear in relation to various situational contexts (Spielberger, 1983). It is widely recognized as a significant impediment to achievement across multiple domains, particularly in educational settings (Seipp, 1991; Steinmayr et al., 2016). The complexity of anxiety research is underscored by the contemporary psychological and neuroscientific distinction between various forms of anxiety, encompassing both general (trait) anxiety (Spielberger, 1983; Spitzer et al., 2006) and specific types, such as math anxiety (Dowker et al., 2016), social anxiety (Pittelkow et al., 2021), and spatial anxiety (Geer et al., 2024). Our study aims to address both general and specific anxieties, such as social and spatial, with a particular focus on anxiety specificity during cognitive problem solving.

General anxiety is often conceptualized as a persistent personality trait, commonly referred to as “trait anxiety,” which predisposes individuals to experience heightened non-specific nervousness and uncontrollable worry over extended periods (Spielberger, 1980; Spitzer et al., 2006). Trait anxiety serves as a framework for understanding individual differences in anxious responses, applicable to both healthy populations (Löwe et al., 2008; Malanchini et al., 2017) and clinical samples (Spitzer et al., 2006). General propensity to experience anxiety symptoms can significantly impair cognitive control, as intrusive worrisome thoughts disrupt task-relevant processing (Pajkossy et al., 2017). This underscores the importance of examining the nuanced interplay between various types of anxiety and their implications for cognitive problem solving.

One of the complexities in anxiety research is the tendency to examine specific anxieties within their respective domains. For example, spatial anxiety is frequently studied in relation to spatial tasks, such as navigation (Lawton, 1994). Research indicates that spatial anxiety has a significant detrimental effect on spatial performance, particularly in tasks involving mental rotation, spatial perception, and spatial visualization (Alvarez-Vargas et al., 2020; Wei et al., 2018). Additionally, the relationship between spatial anxiety and general anxiety measures has been shown to be relatively weak to moderate, with correlation coefficients ranging from 0.24 to 0.44 (Malanchini et al., 2017). Moreover, spatial anxiety impacts cognitive self-evaluation and performance: ERP demonstrated that individuals with high levels of spatial anxiety exhibit increased N2 amplitudes and decreased P3 amplitudes, suggesting that spatial anxiety may influence cognitive strategies and overall cognitive efficiency (Lourenco and Liu, 2023). These findings underscore the importance of understanding the nuanced effects of spatial anxiety on cognitive processes and performance outcomes.

Social anxiety represents another form of anxiety, characterized by significant fear and behavioral inhibition in social contexts (Stein et al., 2017). Research indicates that individuals with elevated social anxiety tend to exhibit lower self-esteem and heightened self-criticism compared to their counterparts with minimal social anxiety (Cox et al., 2004). Furthermore, these individuals demonstrate deficits in cognitive control when processing emotionally neutral stimuli. In participants with high social anxiety, such a neurophysiological marker as the enhanced N2 component of ERP may reflect attentional allocation during congruent trials, suggesting an increased focus on relevant stimuli in the context of conflict monitoring (Cao et al., 2022).

Psychophysiological research, perhaps, could help shedding light on the nature of different anxieties. Several studies have examined neural correlates of anxiety in the presence of disturbing stimuli: processing of emotionally coloured words is influenced by increased overall anxiety, which has been associated with a delayed response to emotionally coloured stimuli in the P3 component (Fisher et al., 2014); individuals with heightened social anxiety exhibit hypersensitivity and difficulties in processing emotionally coloured facial expressions, as evidenced by findings in the N2 component (Hagemann et al., 2016) and the P1 component (Peschard et al., 2013). Also, EEG studies have shown that anxiety symptoms (e.g., uncontrollable worry) are positively correlated with both fronto-occipital interhemispheric alpha-band coherence and occipital and parietal interhemispheric alpha and theta-band coherence (Schoenberg, 2020). Also, the theta-band activity may be a biomarker of anxiety. In this vein, event-related negativity was significantly elevated in participants with anxiety compared to an undiagnosed sample (Hanna et al., 2020). An abnormal theta-band activity has also been found in participants with specific social anxiety (Xing et al., 2017).

ERP research on anxiety during Stroop tasks suggests that attentional control impairments in high trait anxiety individuals are often threat-specific rather than generalised to neutral stimuli such as colour-word pairs (Abdel-Khalek and Alansari, 2004). The spatial Stroop paradigm offers a useful framework for probing reactive control mechanisms during conflict resolution (Spinelli and Lupker, 2024). The Flanker task provides a valid experimental probe for spatial anxiety because it fundamentally engages the spatial attention allocation and conflict monitoring processes that are characteristically disrupted in spatially anxious individuals (Siemann et al., 2018). Critically, the arrow-based version of the Flanker task, while not simulating ecologically valid navigation or mental rotation, reliably captures feature-based and spatial attention shifting—the core cognitive mechanisms implicated in spatial anxiety (Siemann et al., 2018; Lourenco and Liu, 2023). The task’s sensitivity to these mechanisms is indexed by well-established ERP components: the N1 and N2 complex reflects early spatial attention shifts during conflict detection, while the P3 component indexes subsequent context updating and spatial reorientation demands (Siemann et al., 2018; Lourenco and Liu, 2023). Additionally, arrow-based Flanker paradigms have been extensively validated in the anxiety literature for examining error-related processing and attentional control deficits (Imburgio et al., 2020; Keller et al., 2022). These converging lines of evidence provided our *a priori* rationale for expecting spatial anxiety to selectively modulate N2 and P3 amplitudes during the Spatial Flanker task.

Individuals with elevated social anxiety show cognitive control alterations even during processing of emotionally neutral stimuli, as evidenced by N2 and N450 modulations across frontal and central sites (Cao et al., 2022). Time-window analyses reveal enhanced early P3 amplitudes (130–260 ms) at frontal electrodes (F3, Fz, F4, FC3, FCz, and FC4), early posterior negativity (250–320 ms) over occipito-temporal regions (P7, P8, P5, P6, and PO7–PO8), and sustained LPP responses (320–700 ms) at centro-parietal sites (C3, Cz, C4, CP3–CP4, and P3–P4). Notably, neutral words elicited larger early posterior negativity amplitudes, whereas social threat words prompted enhanced LPP responses primarily in low-anxiety individuals suggesting that social anxiety alters emotional processing dynamics for threat-related content via EPN and LPP modulation (Cao et al., 2023). However, it remains unclear whether specific anxiety and general (trait) anxiety exist as distinct constructs, particularly in relation to specific stimuli (e.g., social and spatial tasks). This raises important questions about the interplay between general anxiety and its manifestations in response to particular situations or triggers.

Contemporary transdiagnostic models of anxiety posit that general anxiety reflects a shared vulnerability in top-down attentional control, which may manifest as domain-specific anxieties depending on contextual triggers (Norton and Paulus, 2017). While social and spatial anxiety correlate moderately with trait anxiety (e.g., *r* = 0.35, *p* < 0.01 for spatial and *r* = 0.53, *p* < 0.01 for social), they are frequently studied in isolation, leaving it unclear whether their neural correlates during cognitive tasks reflect domain-specific processing or a generalized attentional-control disruption. The aim of our study was to investigate distinct types of anxiety specifically social anxiety and spatial anxiety—and cognitive performance during problem-solving tasks (in Colour, Social, and Spatial tasks). The study conceptualizes social and spatial anxiety as questionnaire-defined, non-clinical profiles reflecting specific anxiety within cognitive domains, rather than clinically diagnosed conditions. To resolve this theoretical ambiguity, the present study tested two competing hypotheses. H1: if anxiety operates via domain-specific threat processing, we predicted that social anxiety would selectively enhance early (e.g., N170) and LPP ERP components during the Social Stroop task, whereas spatial anxiety would selectively enhance spatial attention (N1 and N2) and conflict-monitoring (P3) components during the Spatial Flanker task. H2: if anxiety reflects a generalized, transdiagnostic deficit in top-down attentional control (Norton and Paulus, 2017), we predicted that both anxiety groups would show common modulation of conflict-monitoring (N2 and N3) and context-updating (P3) components across all three cognitive tasks, regardless of stimulus content. This design allows us to determine whether neural alterations are bound to specific anxiety triggers or reflect a broader, non-specific vulnerability.

## 2 Methods

### 2.1 Participants

Sixty participants (54 females, 6 males; age range: 18–30 years) were recruited via University social networks and divided into three groups based on online anxiety screening: Control (*N* = 20), Social Anxiety (*N* = 20), and Spatial Anxiety (*N* = 20). Group assignment criteria required elevated scores (>1 SD) on a single anxiety measure while remaining within ±1 SD on others (see Supplementary Table S1 for demographics). The study was approved by the University ethical committee (13.11.2023, №118); written informed consent was obtained. Participants received monetary compensation (~19 USD).

The required sample size for this study was determined using G*Power v.3.1.9.7 (Faul et al., 2009). The power analysis indicated that a sample of 20 participants per group, resulting in a total sample size of about 60 participants, would be adequate to detect expected moderate effect sizes (effect size = 0.40) with a significance level (*α*) of 0.05 and a statistical power of 0.80. Recent systematic reviews and meta-analyses of EEG studies have reported that the average sample counted 18.25 (Neo et al., 2023) and 19.69 (Prins et al., 2025), further supporting the feasibility of our sample size.

### 2.2 Procedure

**Figure 1:**
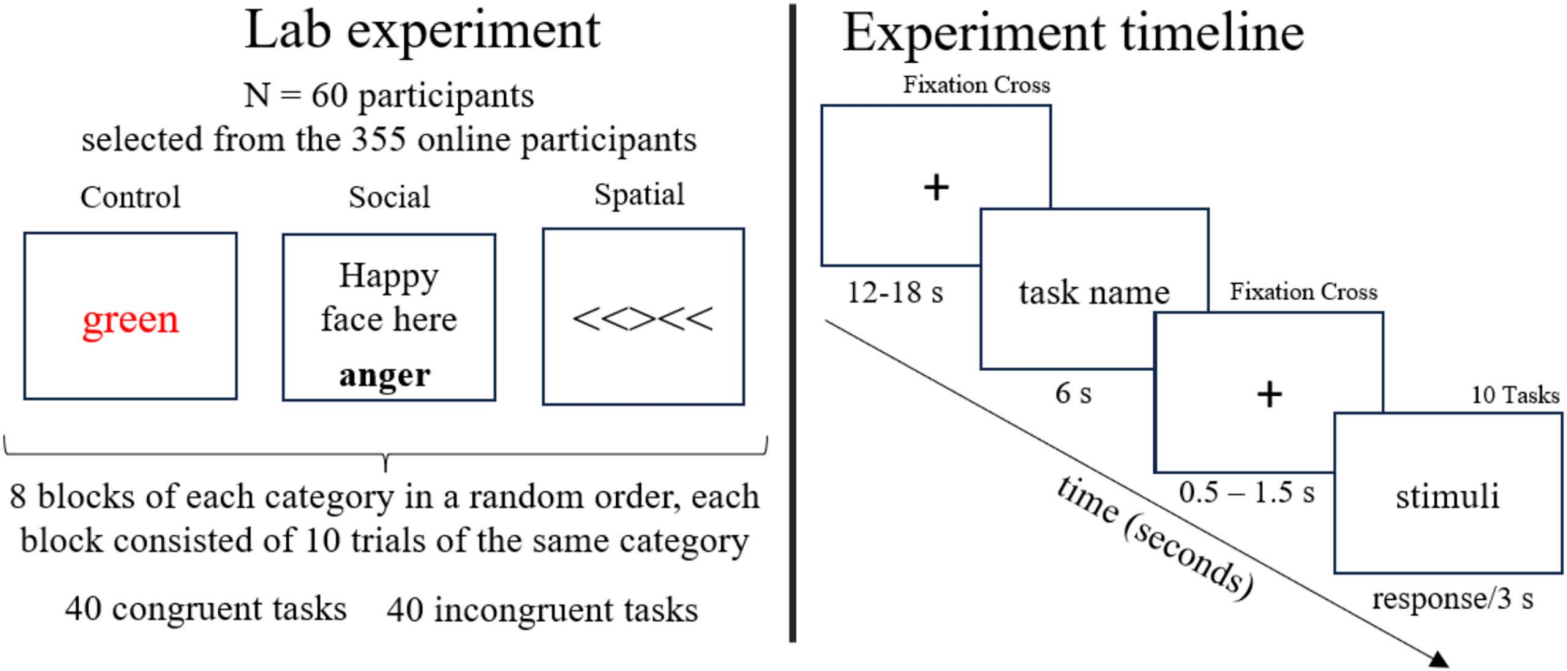
Experiment design. For “stimuli,” if participants do not respond within 3 s, the next stimulus automatically appears.

Participants were recruited online via University social networks and completed an anxiety screening questionnaire (*N* = 355). Based on scores, 60 individuals were selected and assigned to three groups (*N* = 20 each): Control, Social Anxiety, and Spatial Anxiety. Group assignment required elevated scores (>1 SD) on a single anxiety measure while remaining within ±1 SD on all others (see Supplementary Tables S2, S3a-c for thresholds and distributions). The laboratory session (~1.5–2 h) included EEG recording during three cognitive tasks: Colour Stroop, Social Stroop, and Spatial Flanker. Participants completed 80 trials per task in pseudorandom order, with task cues preceding each block and no performance feedback during the main trial. For mode details see Figure 1.

Anxiety levels were assessed using four self-report measures: the Trait subscale of the State–Trait Anxiety Inventory (STAI-T; Spielberger, 1980), the Generalized Anxiety Disorder scale (GAD-7; Spitzer et al., 2006), the Appraisal of Social Concerns questionnaire (ASC; Telch et al., 2004), and the Spatial Anxiety questionnaire (SA; Lawton, 1994). All instruments demonstrated good internal consistency in their adaptations (Likhanov et al., 2026); detailed psychometric properties and scoring procedures are provided in Supplementary Description S1.

Cognitive control was assessed using three conflict tasks: (1) Colour Stroop (neutral control): participants named ink colour of colour words presented in congruent/incongruent ink; (2) Social Stroop: participants identified facial expressions (sad/happy/angry) paired with congruent/incongruent emotion words; (3) Spatial Flanker: participants indicated direction of a central arrow flanked by congruent/incongruent distractor arrows. Each task comprised 80 trials (40 congruent, 40 incongruent) presented in pseudorandom order (see Supplementary Description S1).

### 2.3 Electroencephalography (EEG) recording and preprocessing

EEG data were recorded using the ActiChamp Brain Vision Recorder, employing a configuration of 63 active Ag/Cl electrodes positioned in accordance with the 10–10 system (Koessler et al., 2009). The reference electrode was placed at the Cz location, while the ground electrode was positioned at Fpz. Prior to data acquisition, electrode impedance was carefully monitored and maintained below 5 kΩ to ensure optimal signal quality. The EEG signals were sampled at a frequency of 1,000 Hz, with no online filtering applied during the recording phase. Data preprocessing was conducted using Python, specifically utilizing the MNE library (Gramfort, 2013). Automated visual artifact rejection techniques were implemented to address common issues such as variations in cap placement and body movements, facilitated through the “Autoreject” package (Jas et al., 2017). Epoch rejection by Autoreject had the following parameters: consensus = 0.6, n_interpolate = 3, cv. = 5 (5-fold cross-validation). An epoch was rejected when more than 60% of sensors exceeded the local rejection threshold. Mean rejection rate across participants was 8.2% (SD = 3.1%). Following artifact rejection, bandpass filtering was applied to the data (0.1–30 Hz). After artifact rejection, mean epoch counts per condition were 36–38 trials (less than 10% of the data) across participants. Full trial counts by participant and condition are available in the open dataset. After artifact rejection, mean trial counts per condition were: Colour task (congruent: 37.4 ± 2.1 trials; incongruent: 36.8 ± 2.3), Spatial task (congruent: 38.1 ± 1.9; incongruent: 37.5 ± 2.0), Social task (congruent: 37.9 ± 2.2; incongruent: 37.2 ± 2.4). To further enhance data quality, Independent Component Analysis (ICA) was employed to correct for ocular artifacts associated with eye movements which were recorded with a vertical (put 1 cm below the left eye) and a horizontal (1 cm laterally from the right eye) electrodes. ICA decomposition was performed using the Infomax algorithm with 30 components. Typically 1–2 components per participant were rejected. Bad channels were interpolated via linear interpolation (filling in missing data points in the EEG time series by estimating their values based on the surrounding data with the “raw.interpolate_bads()” function). Within a participant, no more than 7 channels (10%) were interpolated. ERPs were analyzed by creating epochs that spanned from 100 milliseconds prior to the event (baseline) to 1 s after the stimulus onset. Finally, an average signal across all epochs was computed. All data underwent normalization within subjects in preparation for subsequent statistical analyses and visualizations, ensuring consistency and comparability across participants and conditions. ERPs were averaged separately for each combination of task (Colour/Spatial/Social) and congruency (congruent/incongruent). All subsequent statistical analyses were performed on these condition-specific averages.

### 2.4 Data analysis

Behavioral performance (reaction time, accuracy) was analyzed by comparing congruent and incongruent trials. ERP components were defined *a priori* based on established latency and topographical criteria (Luck, 2014): early P3 (150–250 ms) reflecting early sensory processing (e.g., Rezaie et al., 2011); N2 (200–350 ms) associated with conflict monitoring (e.g., Cao et al., 2016); P3 (300–500 ms) indexing context updating (e.g., Brydges and Barceló, 2018); and LPP (400–800 ms) associated with sustained emotional evaluation (e.g., Hajcak et al., 2011). Group differences were assessed using Kruskal-Wallis tests with Dunn’s post-hoc comparisons; Benjamini-Hochberg FDR correction (*q* < 0.05) controlled for multiple comparisons. The FDR correction family was defined separately for each task and included all electrode region and ERP component comparisons (16 tests per task).

## 3 Results

### 3.1 Behavioural results

Behavioral performance was analyzed by calculating the interference effect (incongruent minus congruent) for both reaction time (RT) and accuracy across all three tasks. Descriptive statistics (M, SD, min, max) for these interference effects are provided in Supplementary Tables S4a,b. For additional insights, please see SOM Supplementary Table 5a, which illustrates the correlation between RT interference effects and anxiety questionnaire scores, and SOM Supplementary Table 5b, which depicts the relationship between accuracy interference effects and anxiety scores.

Moreover, non-parametric group analyses using the Kruskal-Wallis test were performed to evaluate potential differences in RT and accuracy interference effects across the three groups. The results indicated no significant group differences in the Colour Task [RT: H(2) = 3.44, *p* = 0.179, p_FDR_ = 0.179, ε^2^ = 0.03; Accuracy: H(2) = 0.95, *p* = 0.622, p_FDR_ = 0.622, ε^2^ = 0.01], Spatial Task [RT: H(2) = 0.79, *p* = 0.674,p_FDR_ = 0.674, ε^2^ = 0.00; Accuracy: H(2) = 2.91, *p* = 0.233, p_FDR_ = 0.233, ε^2^ = 0.02], or Social Task [RT: H(2) = 1.57, *p* = 0.456, p_FDR_ = 0.456, ε^2^ = 0.01; Accuracy: H(2) = 1.72, *p* = 0.423, p_FDR_ = 0.423, ε^2^ = 0.01].

Additionally, a comparative analysis of group performance on congruent and incongruent trials within each task was conducted. The statistical evaluation revealed no significant main effects of group or Group × Congruency interactions for response accuracy or reaction times. These results suggest that behavioral performance was preserved and consistent across participants, irrespective of their anxiety profiles, indicating a homogeneity in task engagement across the sample.

#### 3.1.1 ERPs in colour Stroop task

**Figure 2:**
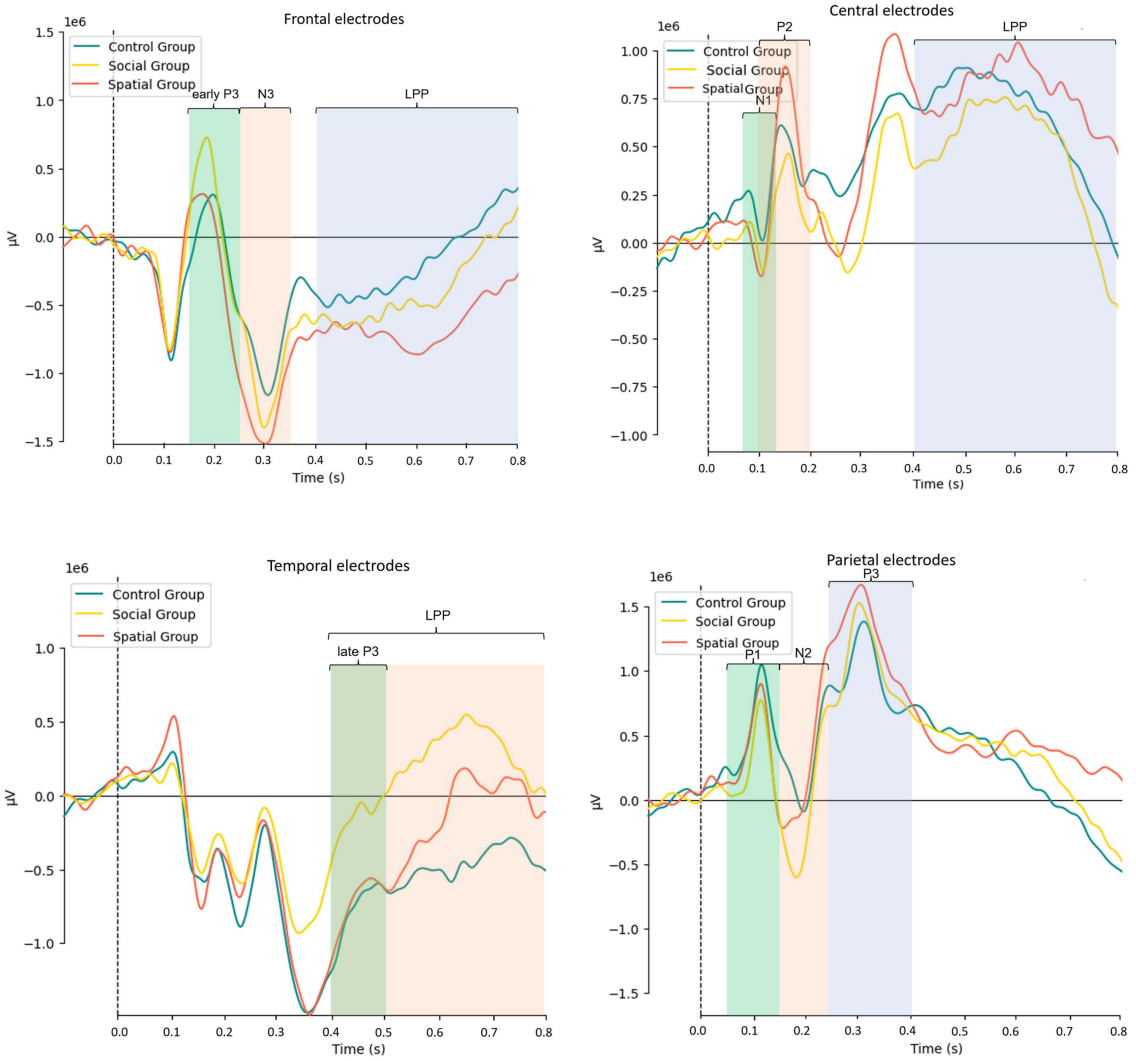
ERPs in the colour task. Soc, social anxiety group; Sp, spatial anxiety group; C, control group.

The analysis of ERP components across various electrode sites revealed significant differences among the three groups (control, social anxiety, and spatial anxiety) during the Colour Task. At frontal electrodes, significant group differences were observed in the amplitudes of early P3 [H(2) = 8.57; *p* = 0.013; ε^2^ = 0.12], N3 [H(2) = 19.77; *p* < 0.001; ε^2^ = 0.31], and LPP [H(2) = 11.90; *p* < 0.001; ε^2^ = 0.17]. After FDR correction (*q* < 0.05), significant effects remained for N3 amplitude at frontal electrodes (p_FDR_ < 0.001) and early P3 amplitude at parietal electrodes (p_FDR_ = 0.008). Other effects reported above did not survive correction and should be interpreted as exploratory. For additional details, please refer to Figure 2 and comprehensive results from the Post-hoc Dunn’s test can be found in SOM Supplementary Tables S6a-d, S7a-d, and SOM Supplementary Figures S1a-d, S2a-d.

#### 3.1.2 ERPs in the spatial task

**Figure 3:**
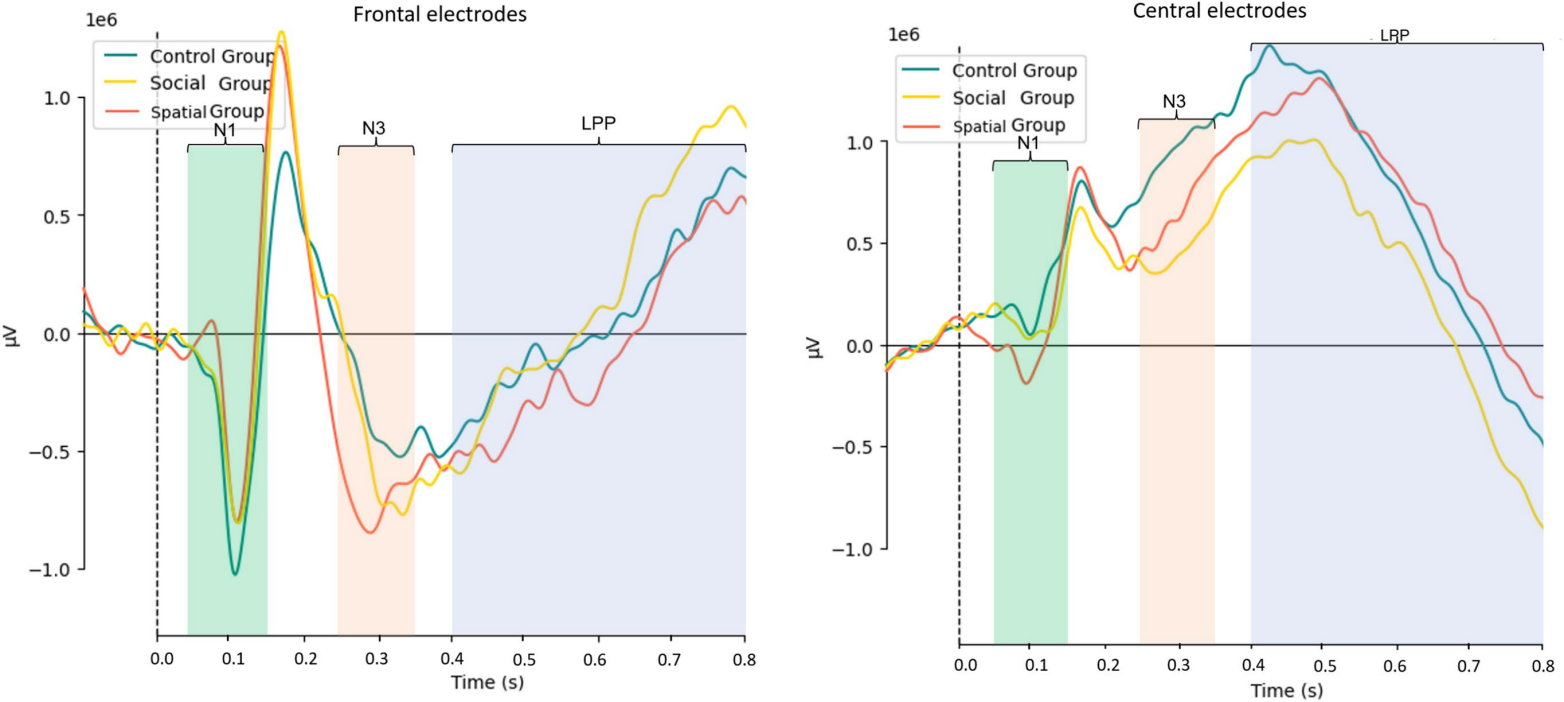
ERPs in the spatial task. Soc, social anxiety group; Sp, spatial anxiety group; C, control group.

The ERP analysis in the Spatial Task revealed significant differences across various components and electrode sites, underscoring the impact of anxiety on cognitive processing. At frontal electrodes, significant effects were observed in the N1 [H(2) = 21.18; *p* < 0.001; ε^2^ = 0.34], N3 [H(2) = 6.76; *p* = 0.034; ε^2^ = 0.08], and LPP [H(2) = 6.20; *p* = 0.045; ε^2^ = 0.07] components. These findings were replicated at central electrode sites, where significant differences were noted in the N1 [H(2) = 11.38; *p* < 0.001; ε^2^ = 0.16], N3 [H(2) = 9.44; *p* = 0.008; ε^2^ = 0.13], and LPP [H(2) = 10.21; *p* = 0.006; ε^2^ = 0.14] components as well. After FDR correction (*q* < 0.05), significant effects remained for N1 and N3 amplitudes at frontal and central electrodes (all p_FDR_ < 0.05). Other effects reported below did not survive correction and should be interpreted as exploratory. For additional insights, see Supplementary Figure S6, which illustrates ERPs in spatial tasks, and refer to Figure 3 for further details on the overall ERP findings. Post-hoc results can be found in SOM Supplementary Tables S8a,b and Supplementary Tables S9a-c, as well as SOM Supplementary Figures S3a,b and Supplementary Figures S4a-c.

#### 3.1.3 ERPs in social Stroop task

**Figure 4:**
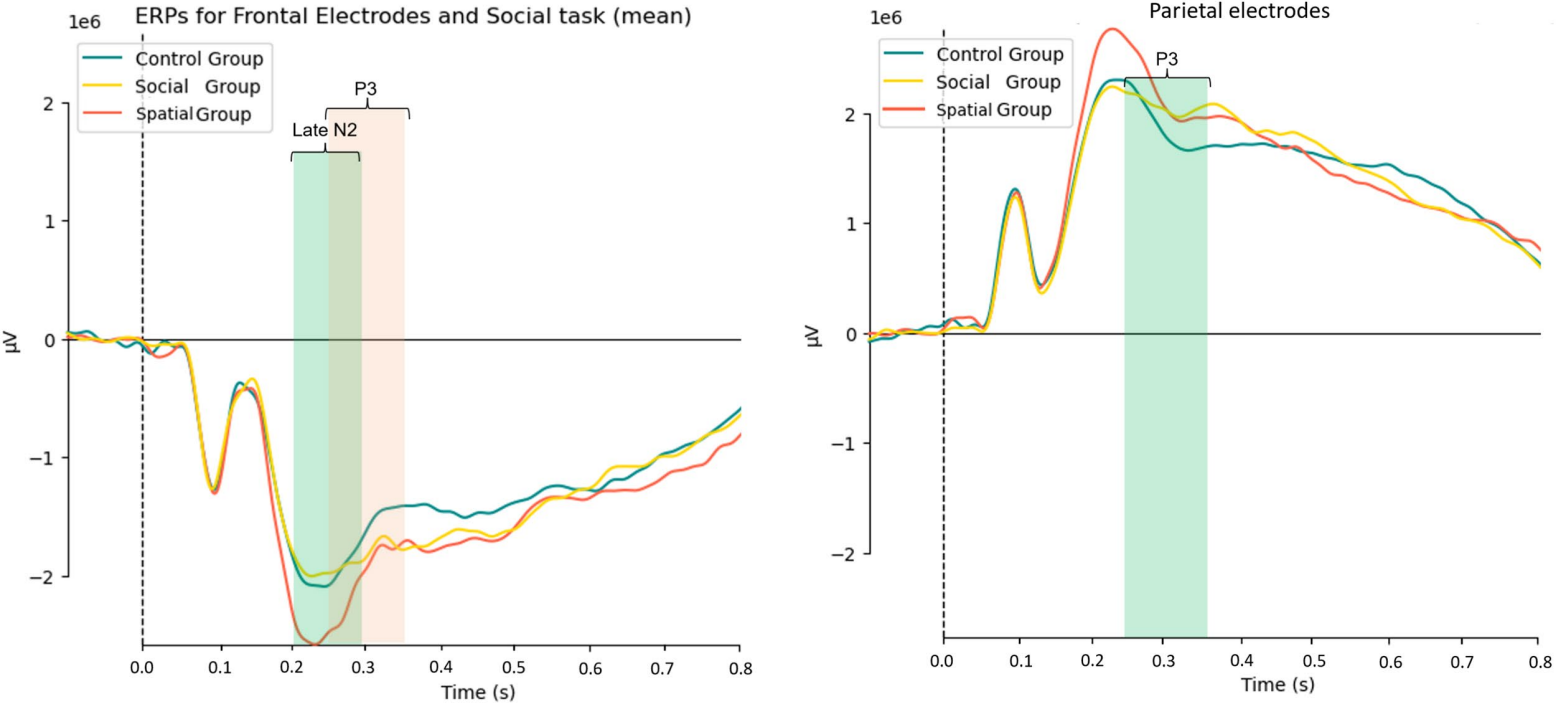
ERPs in social tasks. Soc, social anxiety group; Sp, spatial anxiety group; C, control group.

Significant differences were observed in the Social Stroop Task, particularly in the N2 [H(2) = 14.18; *p* < 0.001; ε^2^ = 0.21] and P3 [H(2) = 16.53; *p* < 0.001; ε^2^ = 0.26] components for frontal electrodes. Notably, a significant effect for the P3 component was also identified at parietal electrodes [H(2) = 14.32; *p* < 0.001; ε^2^ = 0.22]. After FDR correction (*q* < 0.05), significant effects remained the same (all pFDR < 0.05). Other effects reported below did not survive correction and should be interpreted as exploratory. For more details, see Figure 4, and additional information regarding post-hoc analysis can be found in SOM Supplementary Tables S10a,b and Supplementary Tables S11a-d, as well as SOM Supplementary Figures S5a,b and Supplementary Figures S6a-d.

## 4 Discussion

The current study aimed to elucidate different manifestations of anxiety-specifically social and spatial anxiety-and their effects on cognitive processing during three conflict tasks (Colour, Social, and Spatial Stroop). We examined whether domain-specific anxiety profiles would be associated with distinct ERP alterations. In line with prior research by König et al. (2021), no significant behavioral differences in reaction times or response accuracy among the groups was found (König et al., 2021). However, the analysis revealed noteworthy significant findings in the ERP components, suggesting that while overt behavioral measures may not differ, the underlying neural alterations might be influenced by the type of anxiety experienced.

The control group exhibited lower ERP amplitudes for early P3 and N3 at frontal sites compared to anxiety groups. These amplitude differences could reflect differences in neural resource allocation during conflict processing. These findings are in line with evidences of higher local switch costs observed in P3 ERP components compared to low trait anxiety individuals (Wu et al., 2021). Moreover, our findings align with Alansari (2004), who indicated that high trait anxiety does not uniformly affect cognitive tasks; rather, its impact is context-specific (Alansari, 2004).

Social anxiety group consistently displayed medium ERP values, suggesting heightened anxiety levels compared to control, but lower challenges in information processing compared to the spatial anxiety group. This is partially in line with a study by Yin et al. (2024), who found that threat words elicited larger N170 responses than neutral words only in individuals with the low social anxiety; this emotional effect was not observed in those with the high social anxiety (Yin et al., 2024). However, there remains a paucity of literature that explicitly identifies spatial and social anxieties as contributors to impaired cognitive performance in the Colour Stroop task. This gap highlights the need for further investigation.

The spatial anxiety group displayed the highest mean amplitudes for the N1 component in frontal electrodes the same as the social anxiety group, suggesting differences in neural resource allocation during information processing. This finding aligns with existing literature that posits heightened sensitivity to sensory stimuli in anxious individuals, as evidenced by enhanced N1 amplitudes (Weymar et al., 2014). Interestingly, despite these processing challenges, a decreased amplitude for the N3 component was observed, indicating that individuals in both groups may lack the cognitive resources needed to manage information processing under stress (Purmann et al., 2011). Our study also demonstrated significantly lowest amplitude for spatial anxiety group in LPP in frontal electrodes, which is the modulation of late components such as N450 further underscores the role of focused attention in mitigating interference effects during conflict tasks (Galashan et al., 2019).

In summary, participants with elevated anxiety showed enhanced amplitudes in conflict-related ERP components across all three tasks. Critically, these neural differences occurred despite preserved behavioural performance—a pattern consistent with the Processing Efficiency Theory (Eysenck and Calvo, 1992). It is important to note that while effects in the *a priori* conflict-monitoring window (250–350 ms, frontal electrodes) survived FDR correction, several other amplitude modulations did not and should be interpreted as exploratory. While our findings do not definitively establish a shared transdiagnostic mechanism, they are compatible with models positing non-specific disruption of top-down attentional control across anxiety types.

### 4.1 Limitations and future directions

Several limitations should be noted. First, the sample size (*N* = 60; 90% female) and selection criteria (>1 SD on a single anxiety measure) may limit generalizability and introduce selection bias. Future research should consider more gender-balanced samples to examine potential moderating effects of gender. Second, group assignment relied on statistical rather than clinical cutoffs. Although this approach was required to isolate putative domain-specific effects, it might lead to selection bias. Third, the complex design (3 groups × 3 tasks × multiple ERP components) likely reduced power to detect subtle effects, potentially contributing to null findings for domain specificity. Future research should employ larger, gender-balanced samples, divide participants using clinical thresholds, and more demanding paradigms (e.g., dual-task designs, emotional distractors) to better elucidate transdiagnostic versus domain-specific mechanisms of anxiety. Finally, although the arrow-based Flanker task engages spatial attention allocation and conflict monitoring processes relevant to spatial anxiety, it does not simulate real-world navigation or mental rotation. Future research should incorporate more ecologically valid spatial paradigms (e.g., mental rotation tasks, virtual navigation) to better capture the full spectrum of spatial anxiety effects.

## 5 Conclusion

Participants with elevated anxiety showed enhanced ERP amplitudes (~300 ms) across conflict tasks, primarily distinguishing control individuals from both anxiety profiles rather than differentiating between anxiety subtypes. Although FDR-corrected effects point to generalized conflict-monitoring modulation, several reported findings remain exploratory. Consequently, this pattern might suggest non-specific, anxiety-related modulation of attentional control rather than a definitive transdiagnostic mechanism. Future research with larger, gender-balanced samples and ecologically valid paradigms is needed to determine whether these neural signatures reflect stable transdiagnostic vulnerabilities or context-dependent compensatory recruitment.

## Data Availability

The datasets presented in this study can be found in online repositories. The names of the repository/repositories and accession number(s) can be found at: https://osf.io/34cbu/overview.
