# Supplementary material for: The general and specific in anxiety: an ERP study of social and spatial anxiety
Source: Front Hum Neurosci. 2026 Jul 9;20:1837154. doi: 10.3389/fnhum.2026.1837154 (PMC13391859; doi:10.3389/fnhum.2026.1837154)
Supplement: Supplementary file 1 [file Data_Sheet_1.PDF]

Supplementary Materials for The general and specific in anxiety: an ERP study of social and spatial anxiety

Table S1 Sample by Groups

All Participants			
	N	M	SD
Whole Sample	60	22.30	4.35
Males	6	20.83	2.31
Females	54	22.46	4.50
**Social Anxiety Group**			
	N	M	SD
Whole Sample	20	22.35	4.36
Males	2	21.5	2.12
Females	18	22.44	5.57
**Control Group**			
	N	M	SD
Whole Sample	20	22.80	5.91
Males	2	21.50	3.53

Females	18	22.94	6.17

Spatial Anxiety Group

	N	M	SD
Whole Sample	20	21.25	2.04
Males	2	19.50	2.12
Females	18	22	1.94

Notes: N – number of participants; M – Mean; SD – Standard Deviations;

Table S2 Means for Anxiety Questionnaires from previous studies

Questionnaire	Reference	N	M(SD)	The score threshold accepted in our study
Trait Anxiety (STAI-T)	(Zsido et al., 2020)	2227	M(SD) = 44.4 (11.3)	>55.63
Trait Anxiety (GAD-7)	(Löwe et al., 2008)	5030	2.95 (3.41)	>6.34
Social Anxiety (ASC)	(Telch et al., 2004)	N = 550<br>Range 0 - 100	35.59(19.52)	>55.00
		Adapted for range 0-5	1,77 (0,97)	2.75
Spatial Anxiety (SA)	(Lawton, 1994)	N = 288 females<br>N = 138 males	20.35 (5.33) for females<br>18.41 (5.55) for males	>25.65 for females<br>> 23.95 for males
		Mean for the whole sample	19.38 (5.44)	>24.8

Table S3a Descriptive Statistics by Groups: Control Group (N=20)

	Mean	SD	Skewness	Kurtosis	alpha
Trait Anxiety	39.85	8.34	0.11	0.56	0.85
State Anxiety	32.8	6.65	0.37	0.02	0.83
Generalized Anxiety	4.75	2.83	0.33	-1.05	0.74
Social Anxiety	1.94	0.56	0.86	0.48	0.88
Spatial Anxiety	18.1	5.05	-0.22	-1.53	0.76

Table S3b Descriptive Statistics by Groups: Social Anxiety Group (N=20)

	Mean	SD	Skewness	Kurtosis	alpha
Trait Anxiety	49.05	11.17	-0.08	-1.34	0.92
State Anxiety	43.35	12.25	0.54	-0.65	0.94
Generalized Anxiety	8.1	3.55	0.46	0.4	0.75
Social Anxiety	3.34	0.74	-1.16	1.34	0.91
Spatial Anxiety	24.1	6.54	0.07	-0.98	0.81

Table S3c Descriptive Statistics by Groups: Spatial Anxiety Group (N=20)

	Mean	SD	Skewness	Kurtosis	alpha
Trait Anxiety	44.2	7.98	-0.33	-0.29	0.84
State Anxiety	38.4	9.79	0.38	-0.5	0.89
Generalized Anxiety	6	2.34	0.49	-0.68	0.53
Social Anxiety	2.47	0.45	-0.47	-0.68	0.79
Spatial Anxiety	30.6	4.06	0.55	-0.8	0.47

Table 4a Behavioral Data Analysis. Inhibition Cost in Reaction Time.

	M	SD	min	max

Control Group				
Colour Task	-0.08	0.06	-0.21	0.01
Spatial Task	-0.14	0.07	-0.39	-0.06
Social Task	-0.03	0.15	-0.53	0.21
Spatial Anxiety Group				
Colour Task	-0.11	0.05	-0.21	-0.01
Spatial Task	-0.17	0.11	-0.56	-0.05
Social Task	0.02	0.13	-0.25	0.27
Social Anxiety Group				
Colour Task	-0.1	0.07	-0.3	0.03
Spatial Task	-0.14	0.09	-0.36	-0.05
Social Task	0.02	0.11	-0.18	0.36

Table 4b Behavioral Data Analysis. Inhibition Cost Correct responses.

	M	SD	min	max
Control Group				
Colour Task	0.8	1.47	-1	5
Spatial Task	3.2	6.55	-2	29
Social Task	2.4	3.25	0	15
Spatial Anxiety Group				
Colour Task	0.5	0.89	-1	3
Spatial Task	1.9	8.71	-7	37
Social Task	3.1	5.29	-2	18
Social Anxiety Group				
Colour Task	0.55	1.7	-1	6
Spatial Task	1.35	1.57	-1	4
Social Task	1.7	3.1	-2	14

Table S5a Correlation of responses (inhibition cost) and anxiety questionnaires

	Generalised Anxiety	Trait Anxiety	Social Anxiety	Spatial Task	Colour Task	Social Task
Colour Task						.35*
Spatial Task					.17	.07
Social Anxiety				-.25*	-.05	-.06

Trait Anxiety			.60*	-.33*	.14	-.11
Generalised Anxiety		.59*	.37*	-.20	.00	-.19
Spatial Anxiety	.14	.33*	.41*	-.05	.11	.20

Note: * = p < 0.05;

Table S5b Correlation of reaction time (inhibition cost) and anxiety questionnaires

	Social Task	Spatial Task	Spatial Anxiety	Generalised Anxiety	Trait Anxiety	Social Anxiety
Trait Anxiety						.60*
Generalised Anxiety					.59*	.37*
Spatial Anxiety				.14	.33*	.41*
Spatial Task			-.07	.01	.02	-.02
Social Task		-.10	.07	.26*	.26*	.17
Colour Task	.18	-.08	-.13	-.06	-.10	-.28*

Note: *=p < 0.05;

Table S6a Post Hoc Dunn's test for Colour Task: Frontal Electrodes

Variable	Group 1	Group 2	Dunn's (z)	Cohen's d	p-value	r_rb
N300	Control Group	Spatial Group	4.34	1.55	0.00	0.61
N300	Social Group	Spatial Group	2.98	1.22	0.00	0.52
P200	Social Group	Spatial Group	2.87	1.03	0.00	0.45
P400	Control Group	Spatial Group	1.99	0.50	0.05	0.24
LPP	Control Group	Spatial Group	3.43	1.08	0.00	0.47
LPP	Social Group	Spatial Group	2.02	0.64	0.04	0.30

Note: Group 1 and Group 2 represent the compared groups; Dunn's (z) - standardized test statistic; Cohen's d - effect size; r_rb - rank-biserial correlation;

Figure S1a Post Hoc Dunn’s test for Colour Task: Frontal electrodes

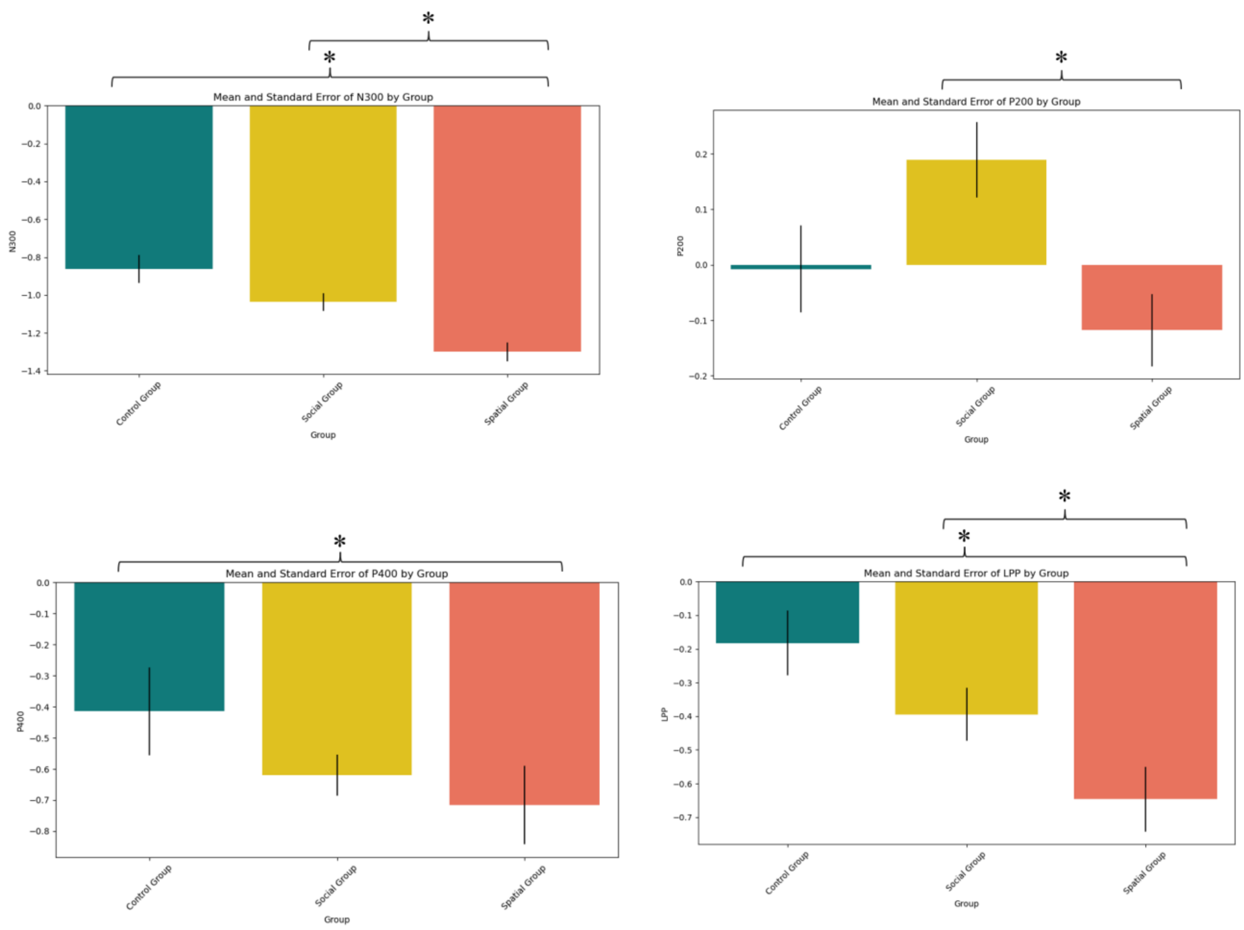

Table S6b Post Hoc Dunn’s test for Colour Task: Central Electrodes

Variable	Group 1	Group 2	Dunn’s (z)	Cohen's d	p-value	r_rb
LPP	Social Group	Spatial Group	-2.68	-1.05	0.01	-0.46
N100	Control Group	Social Group	2.95	1.31	0.00	0.55
early P2	Control Group	Social Group	2.31	0.87	0.02	0.40
early P2	Social Group	Spatial Group	-3.09	-1.01	0.00	0.45
P400	Social Group	Spatial Group	-2.17	-0.79	0.03	0.37

Note: Group 1 and Group 2 represent the compared groups; Dunn’s (z) - standardized test statistic; Cohen's d - effect size; r_rb - rank-biserial correlation;

Figure S1b Post Hoc Dunn’s test for Colour Task: Central electrodes

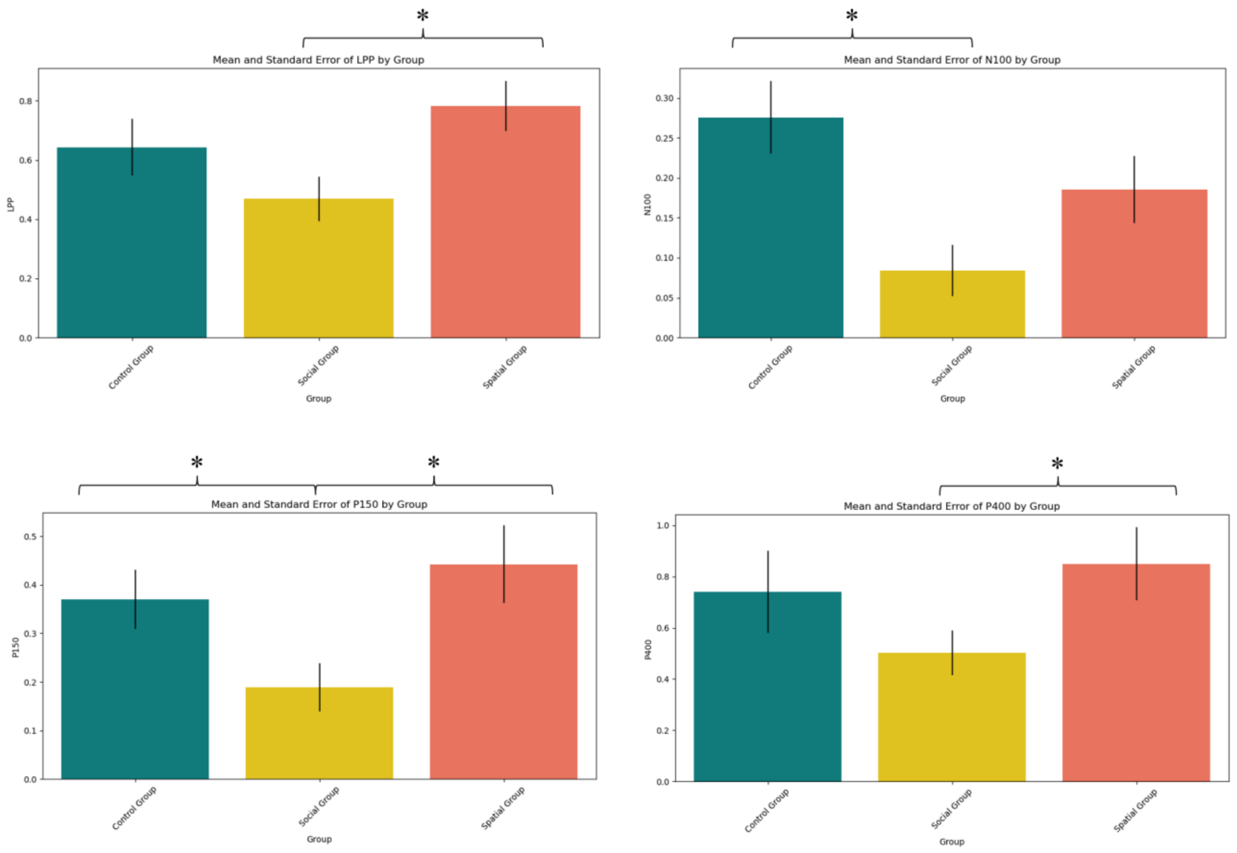

Table S6c Post Hoc Dunn’s test for Colour Task: Temporal Electrodes

Variable	Group 1	Group 2	Dunn’s (z)	Cohen's d	p-value	r_rb
late P3	Control Group	Social Group	-2.32	-1.55	0.02	-0.61
Late P3	Social Group	Spatial Group	-2.32	1.48	0.02	0.59
LPP	Control Group	Social Group	-3.44	-3.12	0.00	-0.84
LPP	Social Group	Spatial Group	2.11	1.48	0.03	0.59

Note: Group 1 and Group 2 represent the compared groups; Dunn’s (z) - standardized test statistic; Cohen's d - effect size; r_rb - rank-biserial correlation;

Figure S1c Post Hoc Dunn's test for Colour Task: Temporal electrodes

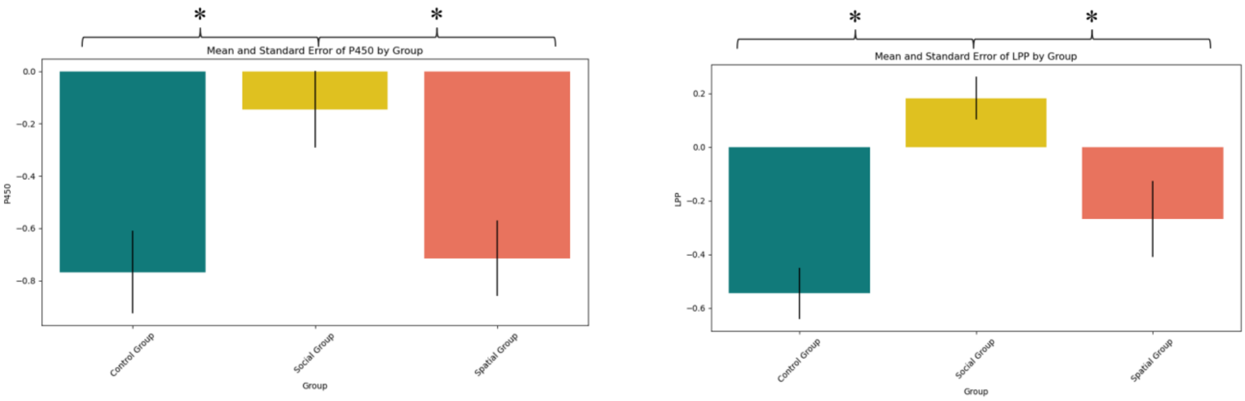

Table S6d Post Hoc Dunn's test for Colour Task: Parietal Electrodes

Variable	Group 1	Group 2	Dunn's (z)	Cohen's d	p-value	r_rb
P100	Control Group	Social Group	4.08	1.80	0.00	0.66
P100	Control Group	Spatial Group	2.04	0.90	0.04	0.40
P100	Social Group	Spatial Group	-2.04	-0.74	0.04	-0.34
N200	Control Group	Social Group	3.61	1.38	0.00	0.56
N200	Social Group	Spatial Group	-3.20	-1.32	0.00	-0.55
P300	Control Group	Spatial Group	-3.12	-1.12	0.00	-0.48
P300	Social Group	Spatial Group	-2.48	-0.96	0.01	-0.43

Note: Group 1 and Group 2 represent the compared groups; Dunn's (z) - standardized test statistic; Cohen's d - effect size; r_rb - rank-biserial correlation;

Figure S1d Post Hoc Dunn’s test for Colour Task: Parietal electrodes

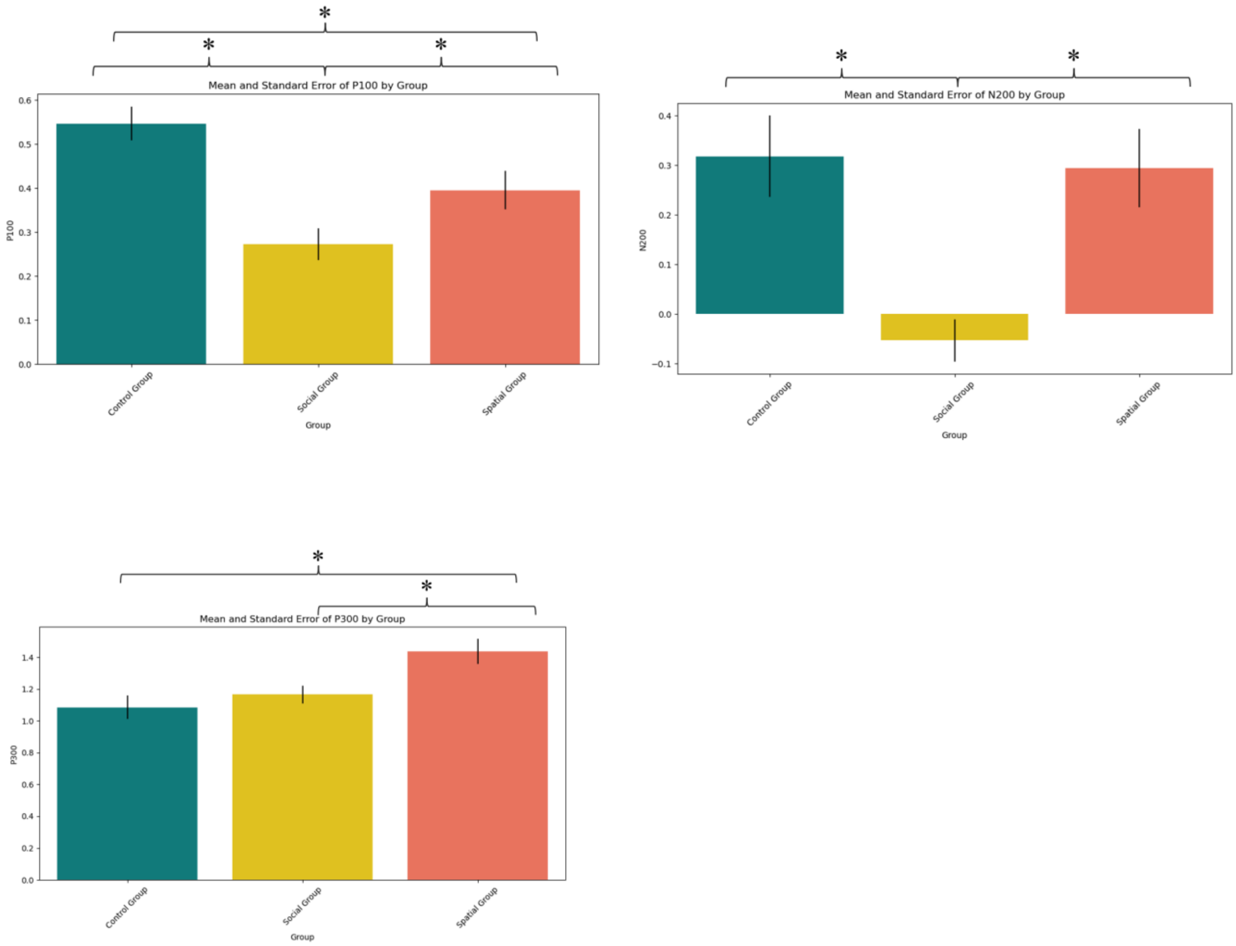

Table S7a Post Hoc Dunn’s test for Colour Task Congruent/Incongruent: Frontal Electrodes

Variable	Group 1	Group 2	Dunn’s (z)	Cohen's d	p-value
		Congruent Task			
N100	Control Group	Social Group	-2.16	-0.54	0.03
P200	Control Group	Social Group	-2.65	-0.81	0.01
LPP	Control Group	Spatial Group	2.47	0.55	0.01
		Incongruent Task			
N100	Control Group	Social Group	-2.73	-0.87	0.01

N100	Social Group	Spatial Group	2.10	0.61	0.04
P200	Control Group	Spatial Group	2.32	0.90	0.02
P200	Social Group	Spatial Group	3.62	1.29	0.00
N300	Control Group	Social Group	2.16	1.05	0.03
N300	Control Group	Spatial Group	5.65	2.22	0.00
N300	Social Group	Spatial Group	3.49	1.72	0.00
P400	Control Group	Spatial Group	2.59	0.65	0.01
LPP	Control Group	Social Group	2.05	0.71	0.04
LPP	Control Group	Spatial Group	4.16	1.51	0.00

Figure S2a Post Hoc Dunn's test for Colour Task Congruent/Incongruent: Frontal Electrodes

Congruent Task

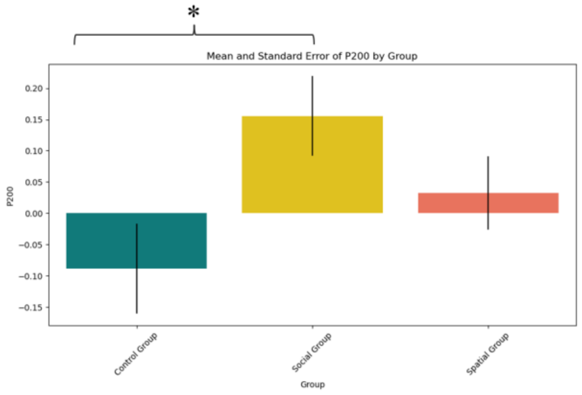

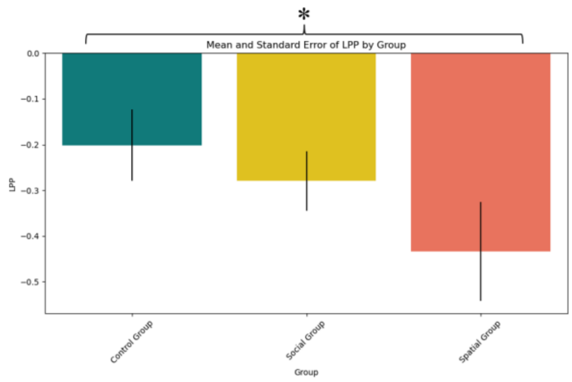

Inongruent Task

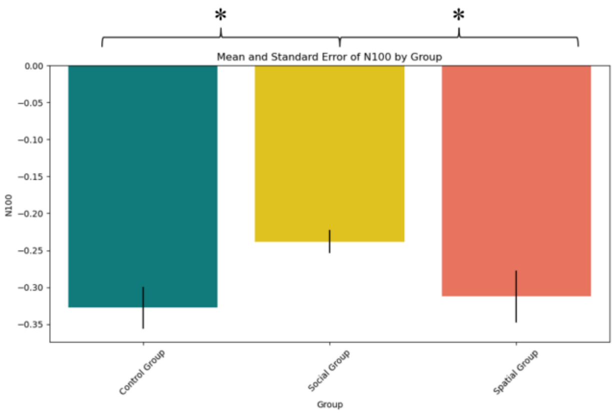

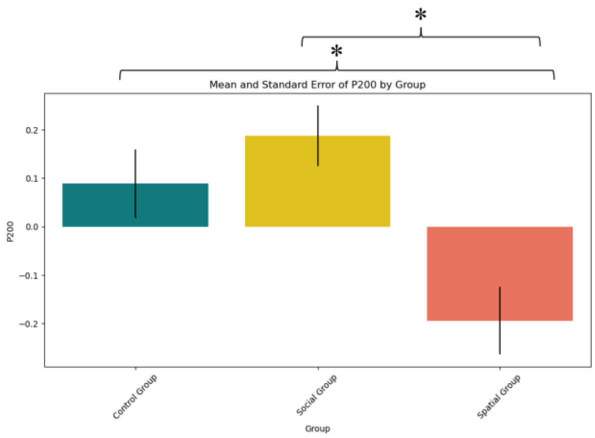

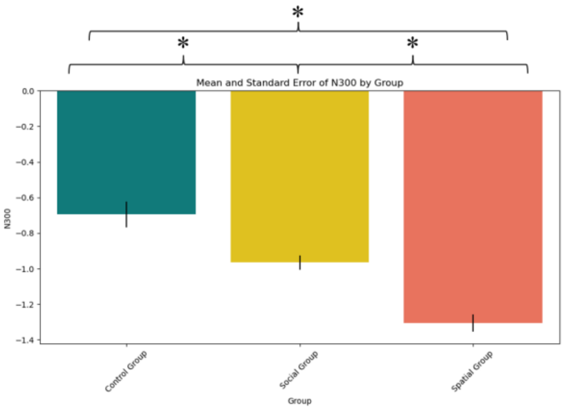

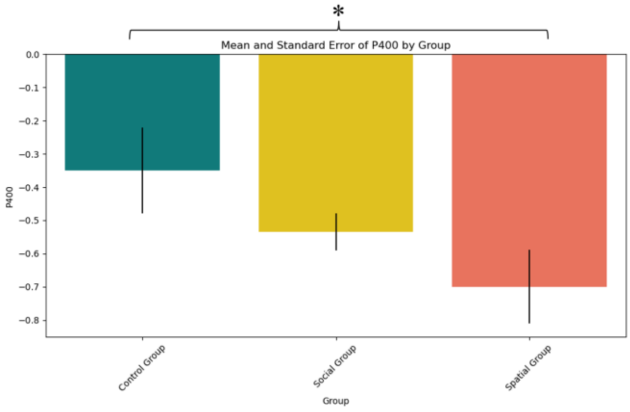

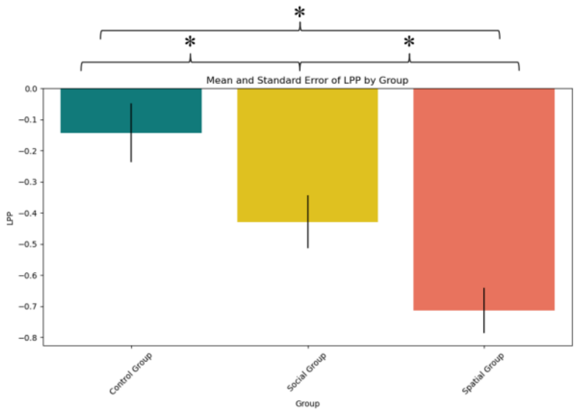

Table S7b Post Hoc Dunn's test for Colour Task Congruent/Incongruent: Central Electrodes

Variable	Group 1	Group 2	Dunn's (z)	Cohen's d	p-value
Congruent Task					
N100	Control Group	Social Group	4.09	2.10	0.00
N100	Social Group	Spatial Group	-2.18	-0.97	0.03
P200	Control Group	Social Group	3.80	1.55	0.00
P200	Social Group	Spatial Group	-2.94	-1.42	0.00
N300	Control Group	Social Group	3.38	0.98	0.02
N300	Social Group	Spatial Group	-3.06	-1.26	0.00

P400	Control Group	Social Group	2.38	0.74	0.02
P400	Social Group	Spatial Group	-3.80	-1.60	0.00
LPP	Control Group	Social Group	2.80	1.33	0.01
LPP	Control Group	Spatial Group	-1.97	-1.02	0.05
LPP	Social Group	Spatial Group	-4.77	-2.50	0.00
Incongruent Task					
None					

Figure S2b Post Hoc Dunn's test for Colour Task Congruent/Incongruent: Central Electrodes

Congruent Task

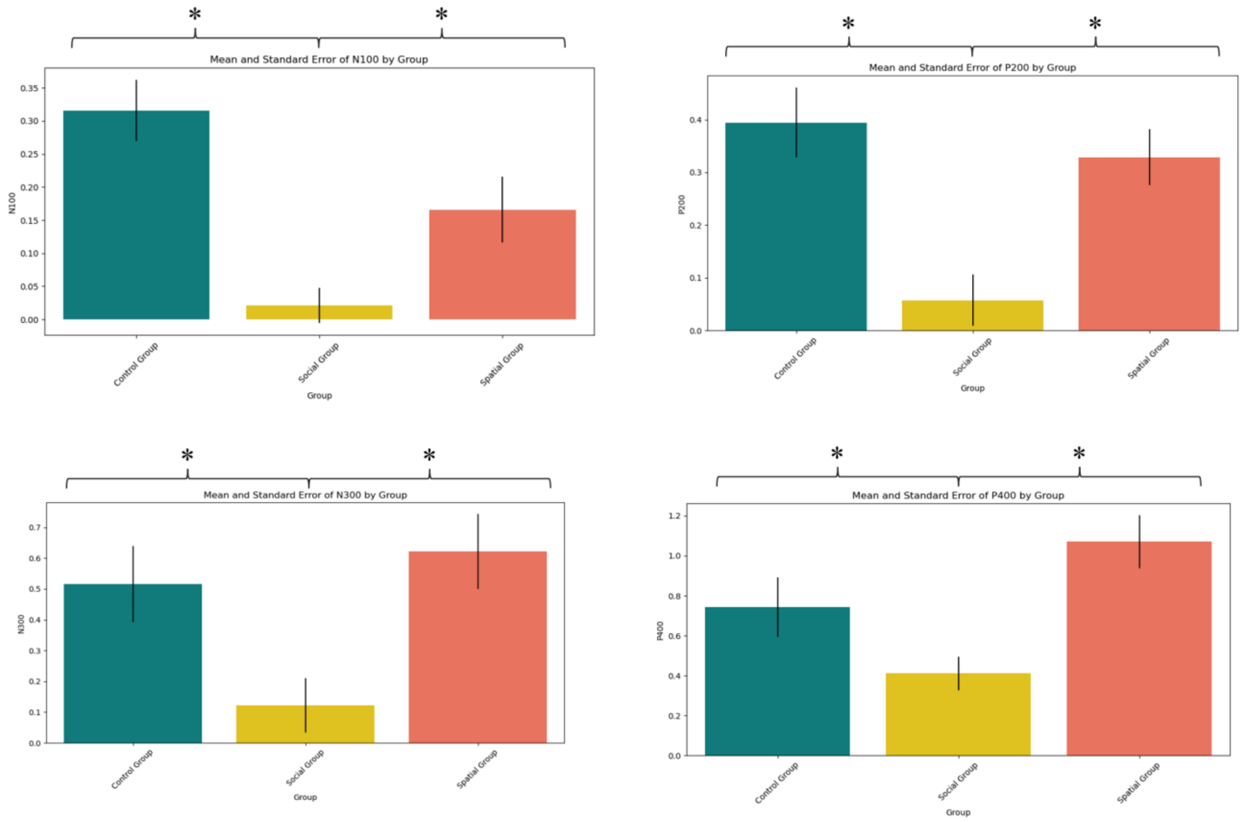

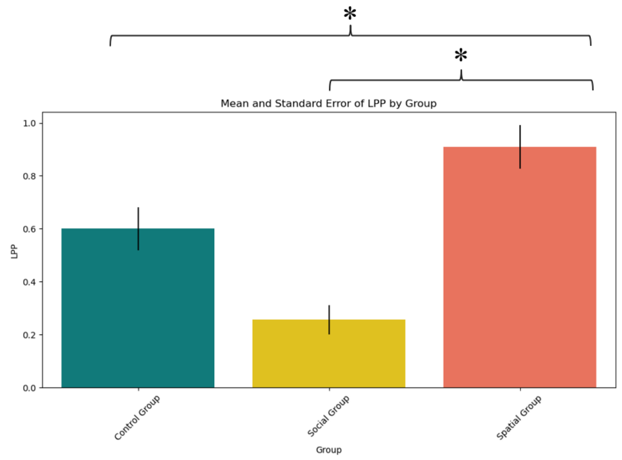

Table S7c Post Hoc Dunn's test for Colour Task Congruent/Incongruent: Temporal Electrodes

Variable	Group 1	Group 2	Dunn's (z)	Cohen's d	p-value
		Congruent Task			
P100	Social Group	Spatial Group	-2.49	-1.67	0.01
N150	Social Group	Spatial Group	-2.06	-1.31	0.04
		Incongruent Task			
N150	Control Group	Social Group	-2.19	-1.46	0.03
P200	Control Group	Social Group	-2.06	-1.39	0.04
N400	Control Group	Social Group	-2.45	-1.83	0.01
N400	Social Group	Spatial Group	2.58	1.75	0.01
LPP	Control Group	Social Group	-3.79	-4.23	0.00
LPP	Social Group	Spatial Group	2.41	2.58	0.02

Figure S2c Post Hoc Dunn’s test for Colour Task Congruent/Incongruent: Temporal Electrodes

Congruent Task

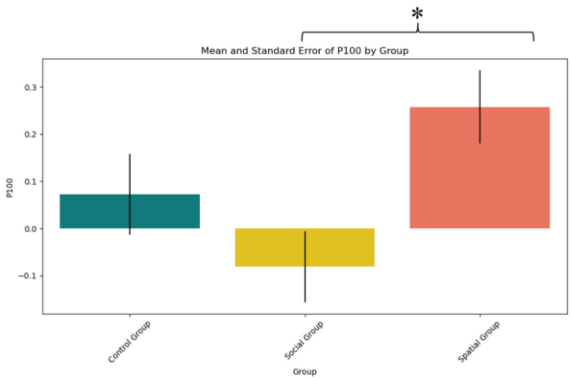

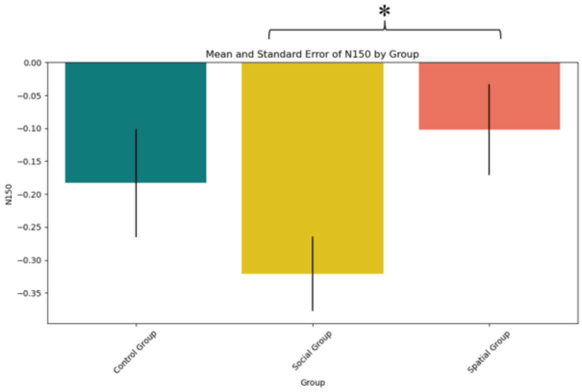

Incongruent Task

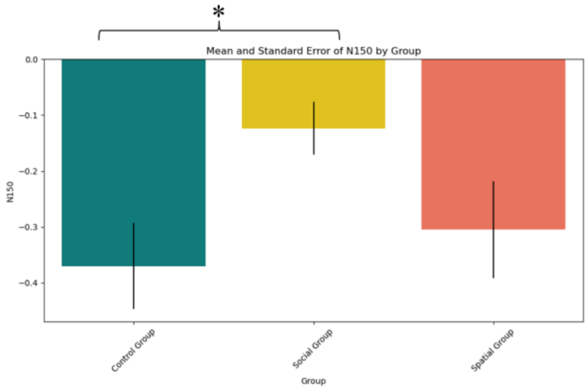

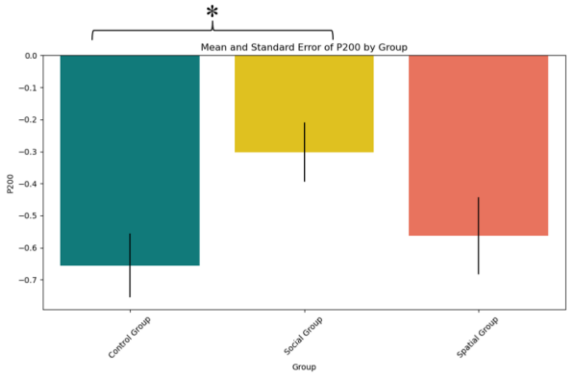

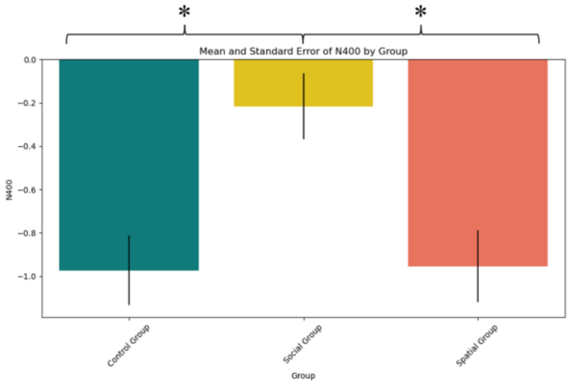

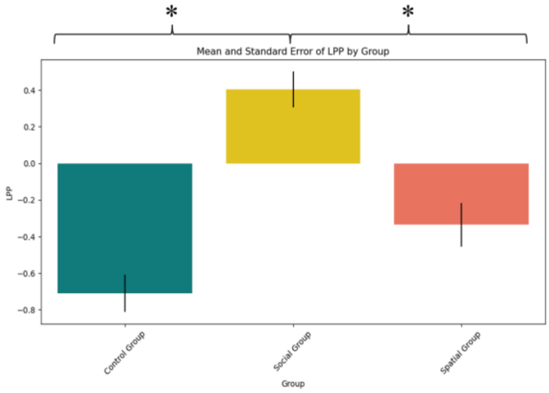

Table S7d Post Hoc Dunn's test for Colour Task Congruent/Incongruent: Parietal Electrodes

Variable	Group 1	Group 2	Dunn's (z)	Cohen's d	p-value
		Congruent Task			
P100	Control Group	Social Group	4.41	2.11	0.00
P100	Social Group	Spatial Group	-3.06	-1.30	0.00
N200	Control Group	Social Group	4.60	2.05	0.00
N200	Social Group	Spatial Group	-3.80	-1.76	0.00
P300	Control Group	Spatial Group	-3.41	-1.47	0.00
P300	Social Group	Spatial Group	-3.81	-1.69	0.00
LPP	Control Group	Spatial Group	-2.25	-0.90	0.02
LPP	Social Group	Spatial Group	-3.35	-1.41	0.00
		Incongruent Task			
P100	Control Group	Social Group	2.96	1.24	0.00
P100	Control Group	Spatial Group	2.15	0.95	0.03
N200	Control Group	Social Group	1.96	0.63	0.05
N200	Social Group	Spatial Group	-2.64	-0.93	0.01
P300	Control Group	Social Group	-2.72	-1.03	0.01

P300	Control Group	Spatial Group	-3.47	-1.21	0.00
LPP	Control Group	Social Group	-2.26	-0.84	0.03

Figure S2d Post Hoc Dunn's test for Colour Task Congruent/Incongruent: Parietal Electrodes

Congruent Task

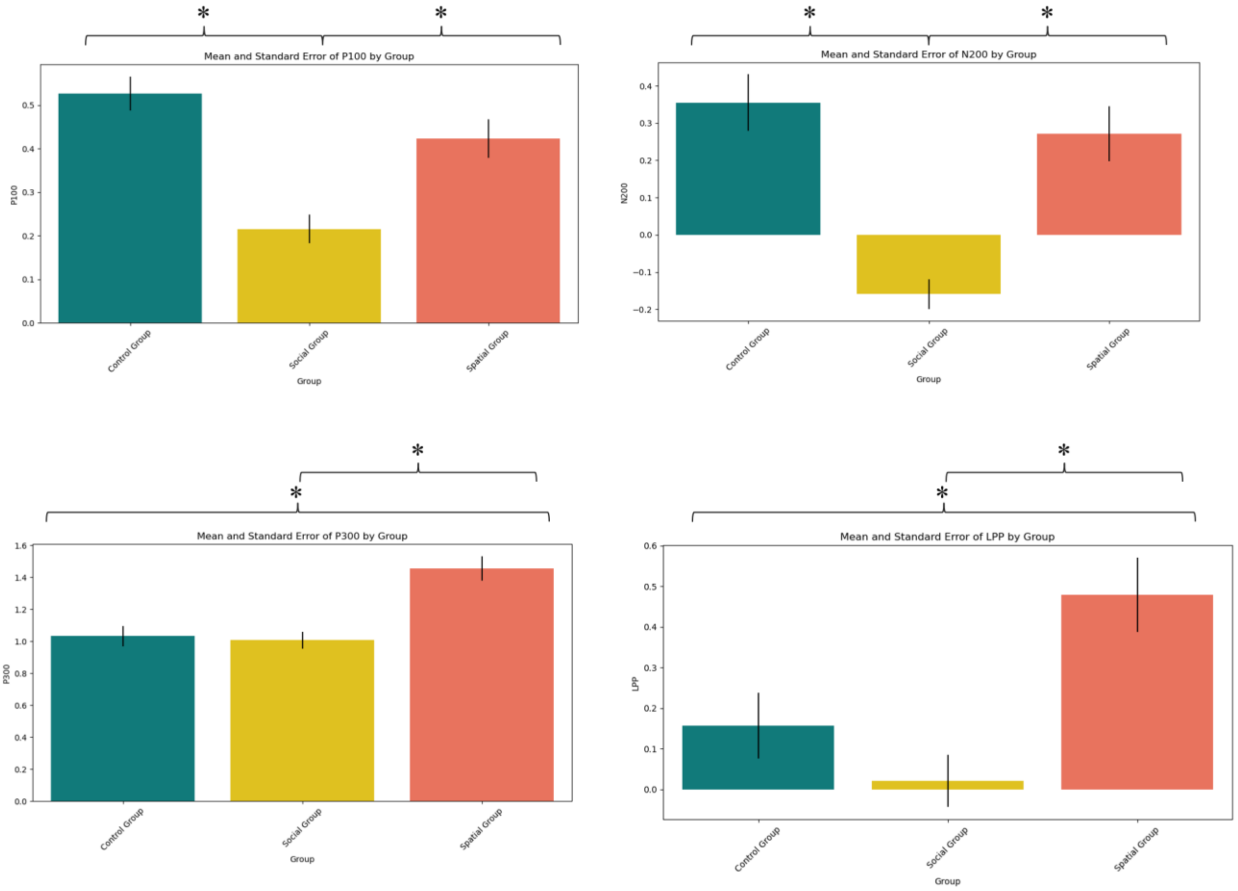

Incongruent Task

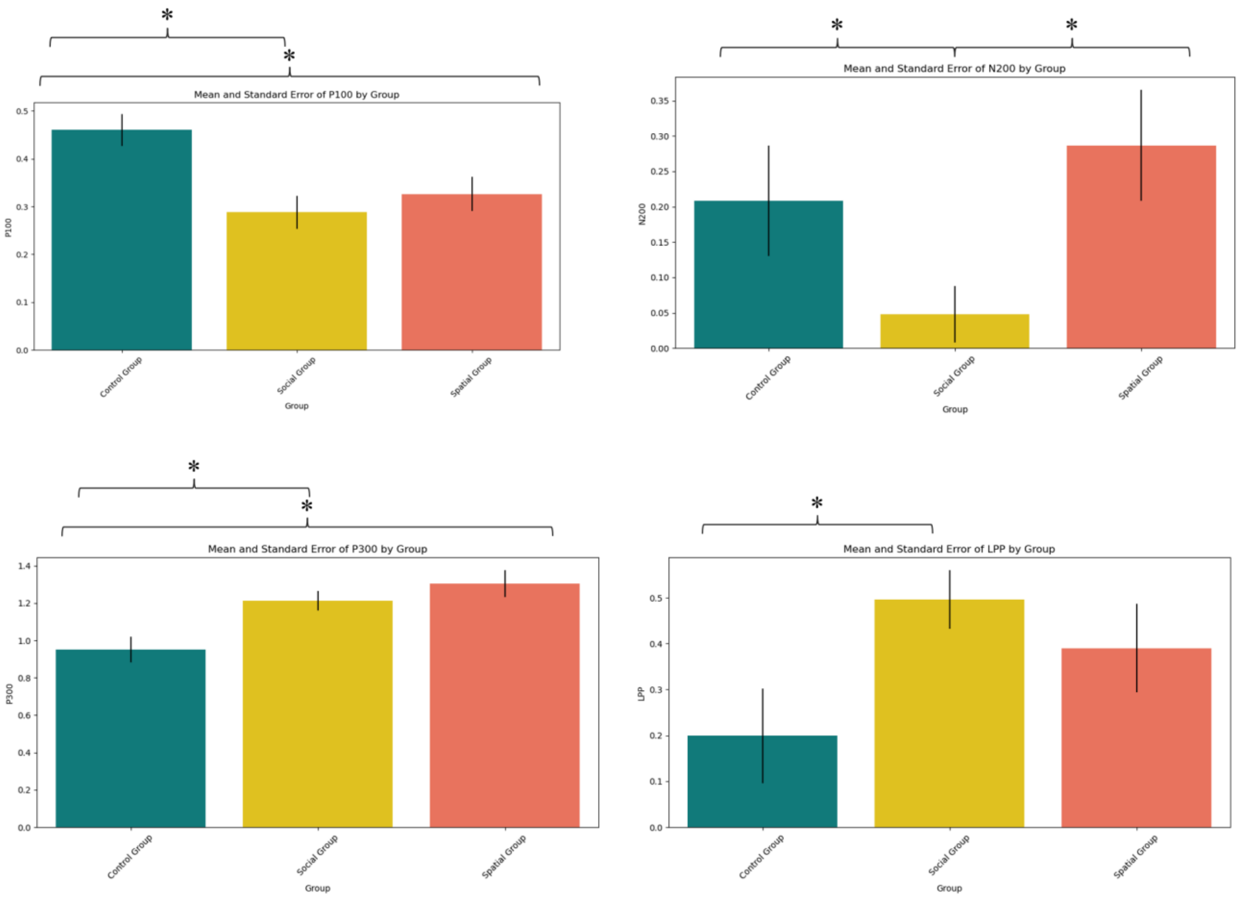

Table S8a Post Hoc Dunn's test for Spatial Task: Frontal Electrodes

Variable	Group 1	Group 2	Dunn's (z)	Cohen's d	p-value	r_rb
N100	Control Group	Spatial Group	-4.58	-1.87	0.00	-0.68
N100	Social Group	Spatial Group	-2.67	-1.35	0.01	-0.55
N300	Control Group	Spatial Group	2.49	0.84	0.01	0.38
LPP	Social Group	Spatial Group	2.44	0.72	0.01	0.33

Note: Group 1 and Group 2 represent the compared groups; Dunn's (z) - standardized test statistic; Cohen's d - effect size; r_rb - rank-biserial correlation;

Figure S3a Post Hoc Dunn’s test for Spatial Task: Frontal electrodes

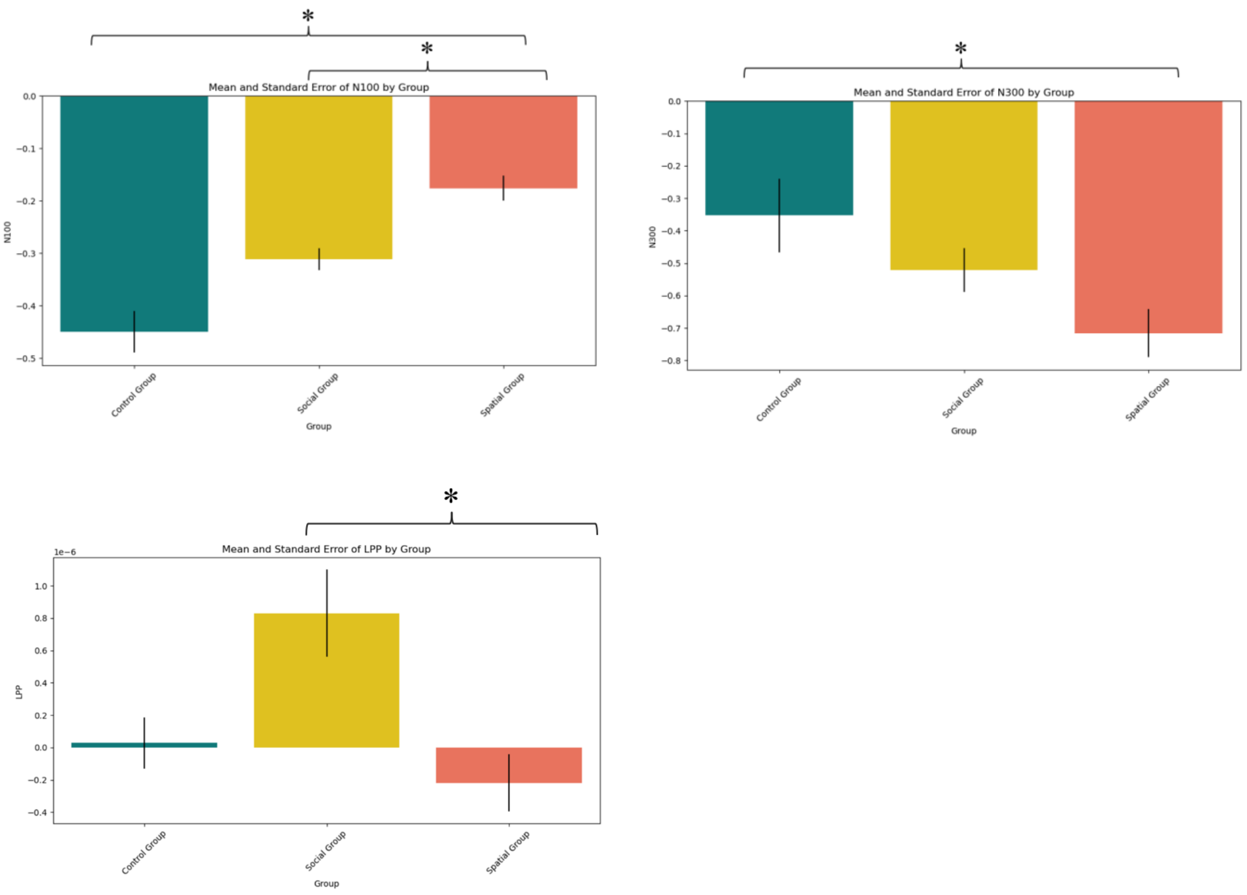

Table S8b Post Hoc Dunn’s test for Spatial Task: Central Electrodes

Variable	Group 1	Group 2	Dunn’s (z)	Cohen's d	p-value	r_rb
N100	Control Group	Spatial Group	3.37	1.39	0.00	0.57
N300	Control Group	Social Group	3.05	1.20	0.00	0.51
LPP	Control Group	Social Group	2.57	1.06	0.01	0.46
LPP	Social Group	Spatial Group	-2.92	-1.19	0.00	-0.51

Note: Group 1 and Group 2 represent the compared groups; Dunn’s (z) - standardized test statistic; Cohen's d - effect size; r_rb - rank-biserial correlation;

Figure S3b Post Hoc Dunn’s test for Spatial Task: Central electrodes

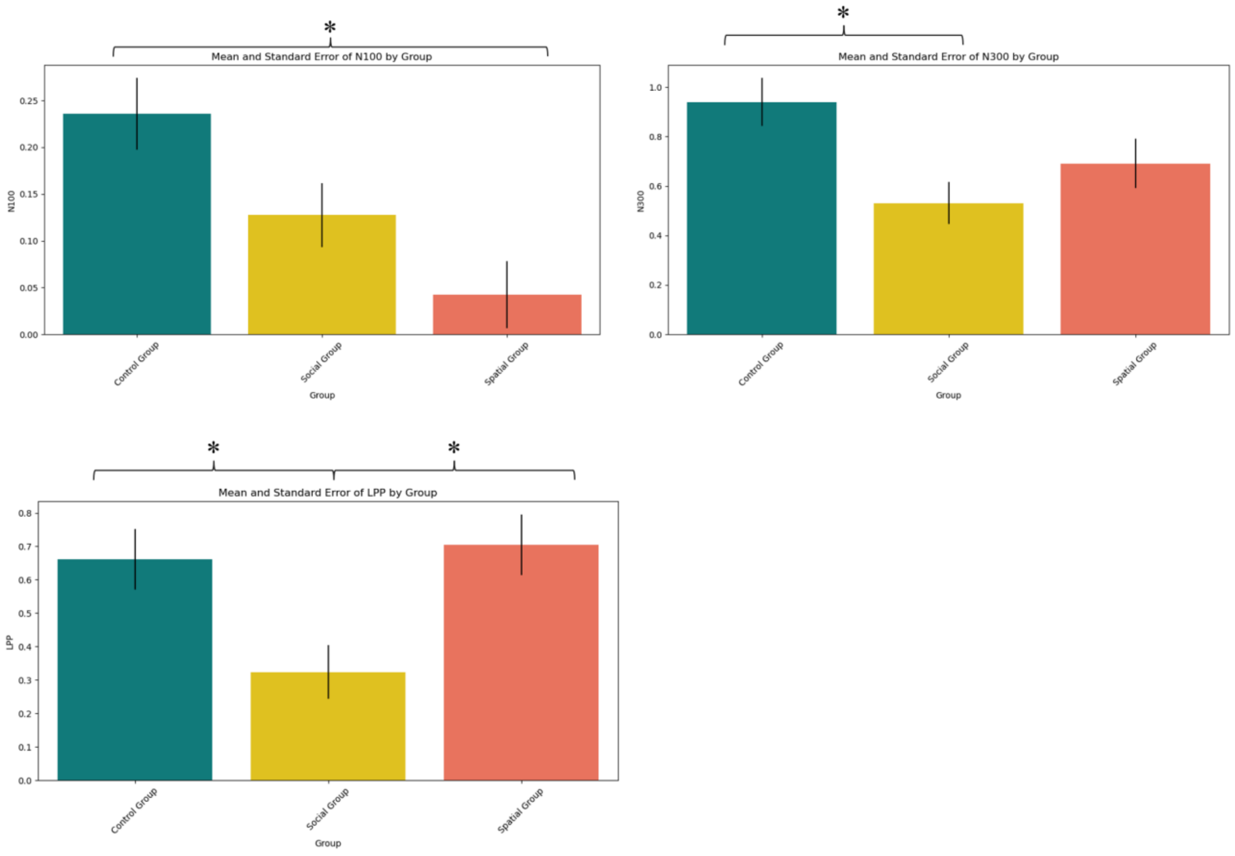

Table S9a Post Hoc Dunn’s test for Spatial Task Congruent/Incongruent: Frontal electrodes

Variable	Group 1	Group 2	Dunn’s (z)	Cohen's d	p-value
Congruent Task					
N100	Control Group	Social Group	-2.47	-1.15	0.01
N100	Control Group	Spatial Group	-5.21	-2.12	0.00
N100	Social Group	Spatial Group	-2.74	-1.31	0.01
N300	Social Group	Spatial Group	2.07	0.64	0.04
LPP	Social Group	Spatial Group	2.19	0.67	0.03
N100	Control Group	Social Group	-2.47	-1.15	0.01

N100	Control Group	Spatial Group	-5.21	-2.12	0.00
N100	Social Group	Spatial Group	-2.74	-1.31	0.01
Incongruent Task					
N100	Control Group	Social Group	-1.97	-1.00	0.05
N100	Control Group	Spatial Group	-4.13	-1.51	0.00
N100	Social Group	Spatial Group	-2.16	-0.97	0.03
N300	Control Group	Spatial Group	2.94	0.97	0.00
N300	Social Group	Spatial Group	2.21	0.74	0.03
LPP	Social Group	Spatial Group	2.84	0.88	0.00

Figure S4a Post Hoc Dunn's test for Spatial Task: Frontal electrodes

Congruent Task

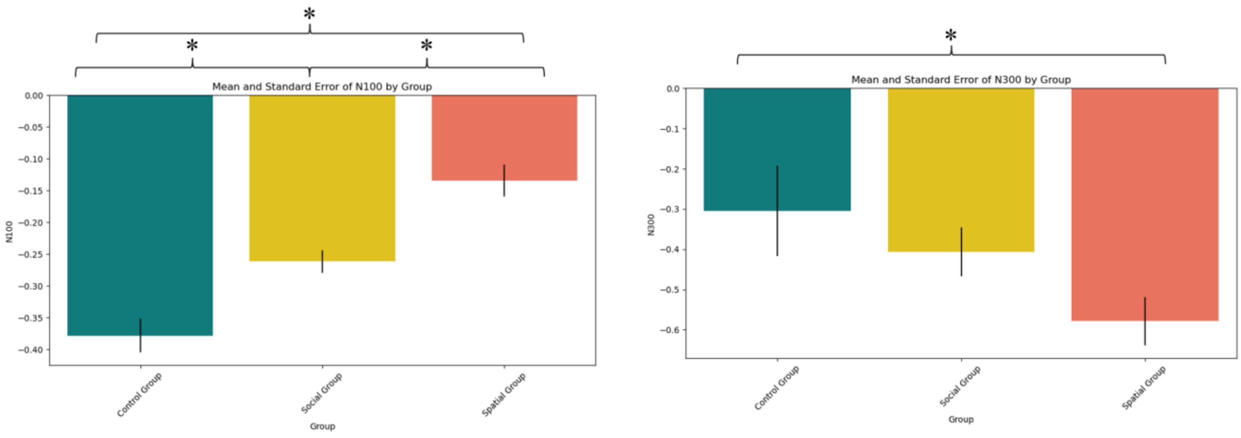

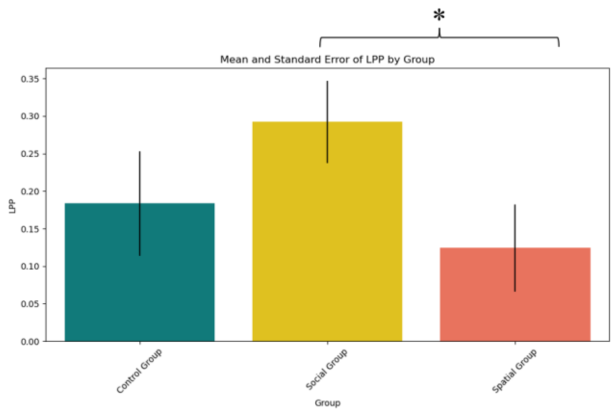

Incongruent Task

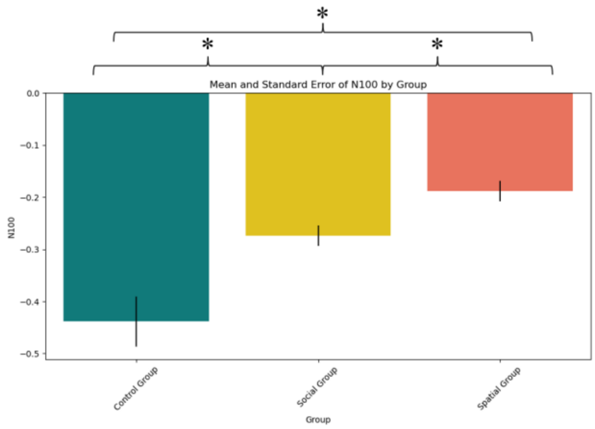

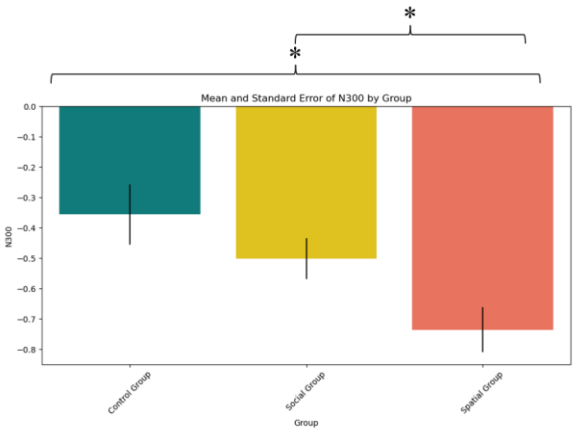

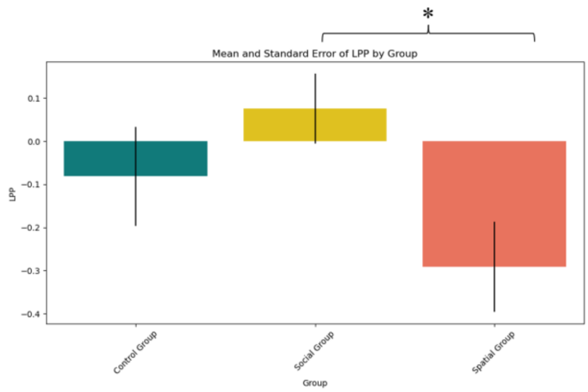

Table S9b Post Hoc Dunn's test for Spatial Task Congruent/Incongruent: Central electrodes

Variable	Group 1	Group 2	Dunn's (z)	Cohen's d	p-value
Congruent Task					

N100	Control Group	Spatial Group	2.26	0.89	0.02
N300	Control Group	Social Group	2.41	0.79	0.02
LPP	Social Group	Spatial Group	-2.20	-0.72	0.03
Incongruent Task					
N100	Control Group	Social Group	2.63	1.27	0.01
N100	Control Group	Spatial Group	4.34	2.00	0.00
N300	Control Group	Social Group	3.58	1.80	0.00
N300	Control Group	Spatial Group	2.69	1.20	0.01
LPP	Control Group	Social Group	2.57	1.19	0.01
LPP	Social Group	Spatial Group	-2.74	-1.23	0.01

Figure S4b Post Hoc Dunn's test for Spatial Task: Central electrodes

Congruent Task

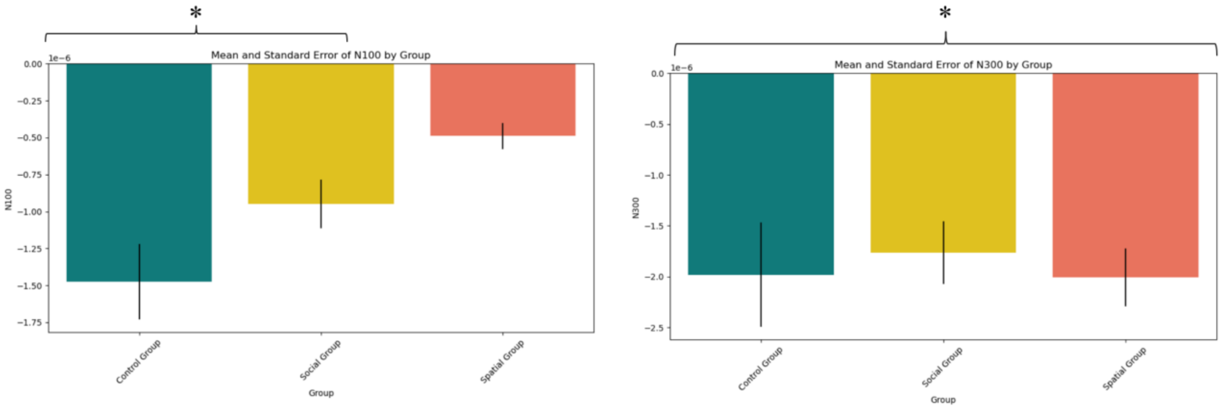

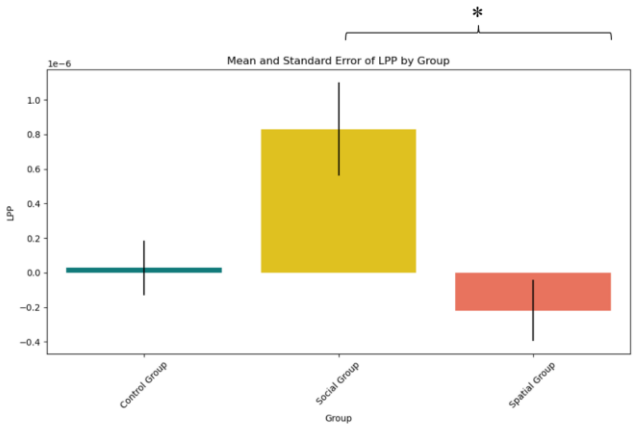

Incongruent Task

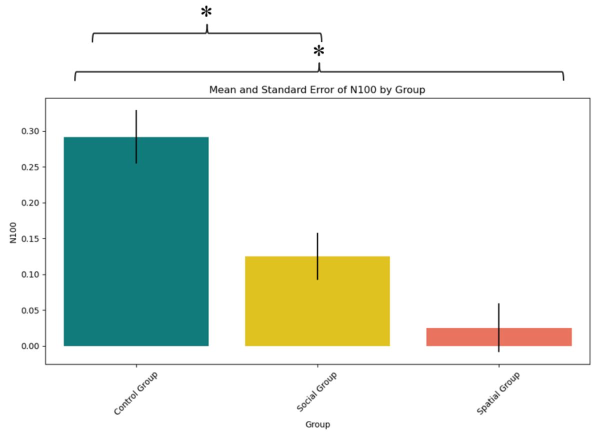

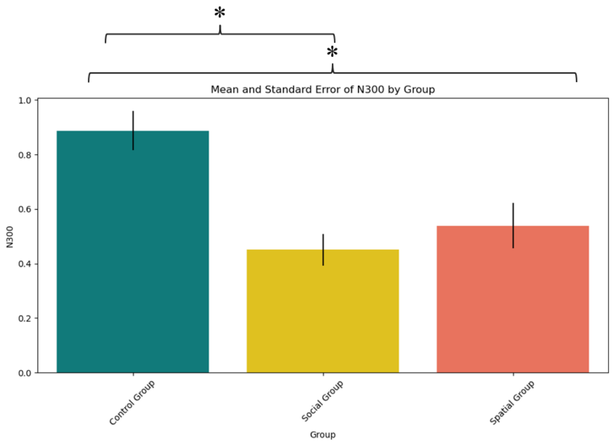

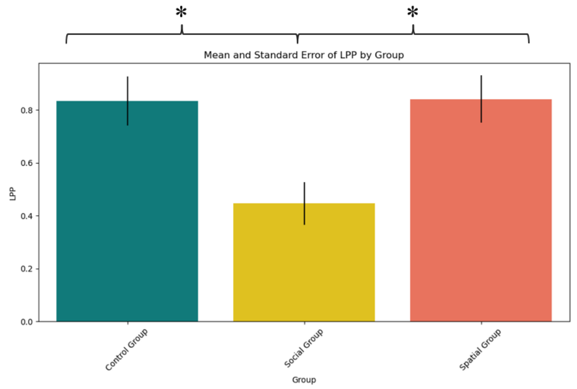

Table S9c Post Hoc Dunn’s test for Spatial Task Congruent/Incongruent: Parietal electrodes

Variable	Group 1	Group 2	Dunn’s (z)	Cohen's d	p-value
Congruent Task					

P100	Control Group	Social Group	3.10	1.67	0.00
P100	Control Group	Spatial Group	5.59	2.48	0.00
P100	Social Group	Spatial Group	2.44	1.33	0.01
		Incongruent Task			
P100	Control Group	Social Group	3.72	2.39	0.00
P100	Control Group	Spatial Group	5.89	3.13	0.00
P100	Social Group	Spatial Group	2.16	1.20	0.03
LPP	Control Group	Social Group	2.00	0.80	0.04

Figure S4c Post Hoc Dunn's test for Spatial Task: Central electrodes

Congruent Task

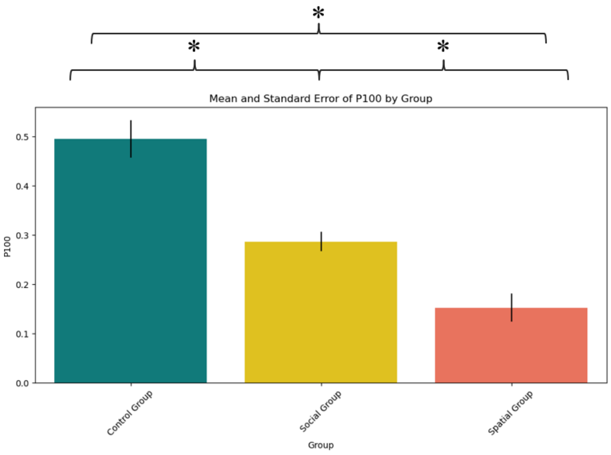

Incongruent Task

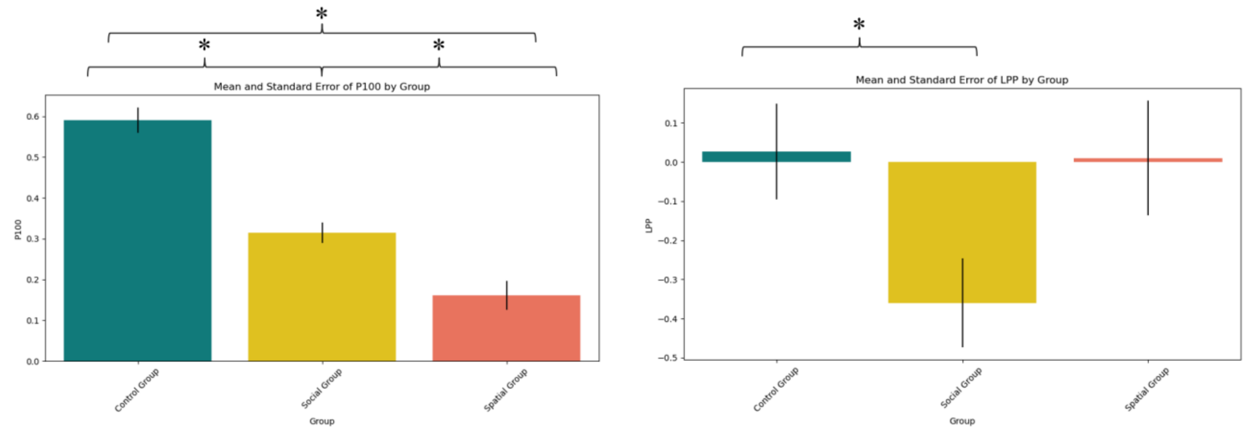

Table S10a Post Hoc Dunn’s test for Social Task: Frontal Electrodes

Variable	Group 1	Group 2	Dunn’s (z)	Cohen's d	p-value	r_rb
N230	Control Group	Spatial Group	3.20	1.22	0.00	0.52
N230	Social Group	Spatial Group	3.31	1.15	0.00	0.49
P300	Control Group	Spatial Group	4.05	1.61	0.00	0.62
P300	Social Group	Spatial Group	2.27	0.74	0.02	0.34

Note: Group 1 and Group 2 represent the compared groups; Dunn’s (z) - standardized test statistic; Cohen's d - effect size; r_rb - rank-biserial correlation;

Figure S5a Post Hoc Dunn’s test for Social Task: Frontal electrodes

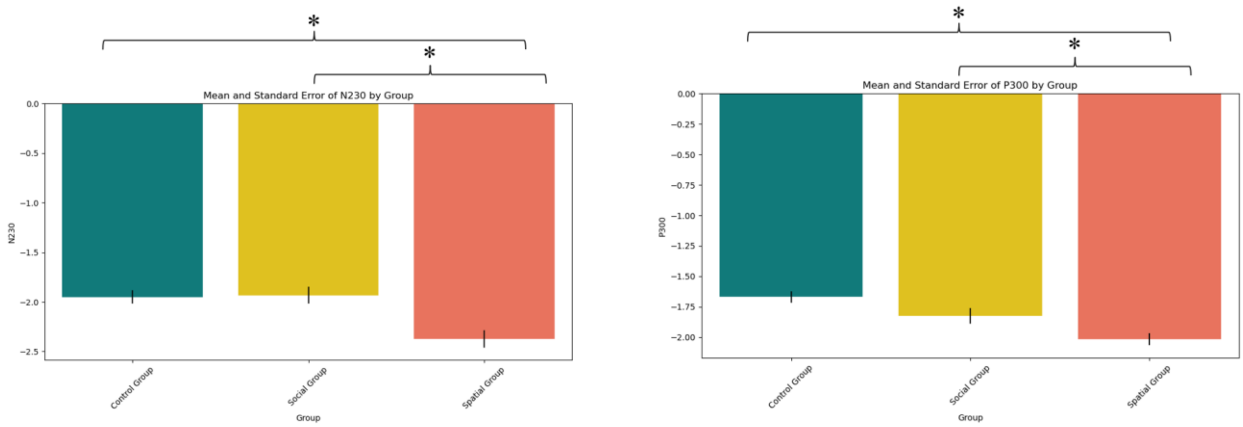

Table S10b Post Hoc Dunn’s test for Social Task: Parietal Electrodes

Variable	Group 1	Group 2	Dunn’s (z)	Cohen's d	p-value	r_rb
P300	Control Group	Spatial Group	-3.78	-1.54	0.00	-0.61

Note: Group 1 and Group 2 represent the compared groups; Dunn’s (z) - standardized test statistic; Cohen's d - effect size; r_rb - rank-biserial correlation;

Figure S5b Post Hoc Dunn’s test for Social Task: Parietal electrodes

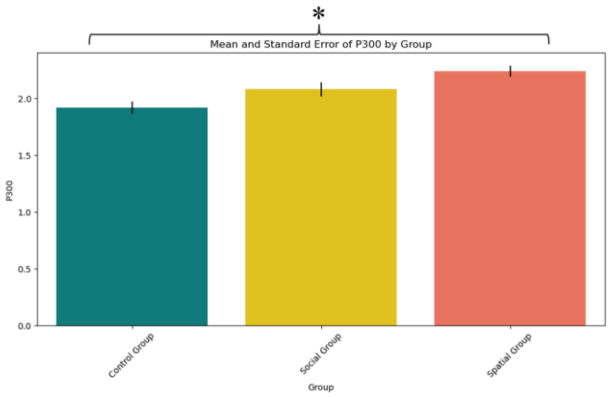

Table S11a Post Hoc Dunn’s test for Social Task Congruent/Incongruent: Frontal electrodes

Variable	Group 1	Group 2	Dunn’s (z)	Cohen's d	p-value
Congruent Task					
N230	Control Group	Spatial Group	3.01	1.14	0.00
N230	Social Group	Spatial Group	2.90	1.01	0.00
P300	Control Group	Spatial Group	3.82	1.57	0.00
LPP	Control Group	Spatial Group	2.43	0.90	0.01
LPP	Social Group	Spatial Group	3.10	1.18	0.00

Incongruent Task					
N230	Control Group	Spatial Group	3.35	1.26	0.00
N230	Social Group	Spatial Group	3.76	1.37	0.00
P300	Control Group	Spatial Group	4.03	1.41	0.00
P300	Social Group	Spatial Group	2.12	0.66	0.03

Figure S6a Post Hoc Dunn's test for Social Task Congruent/Incongruent: Frontal Electrodes

Congruent Task

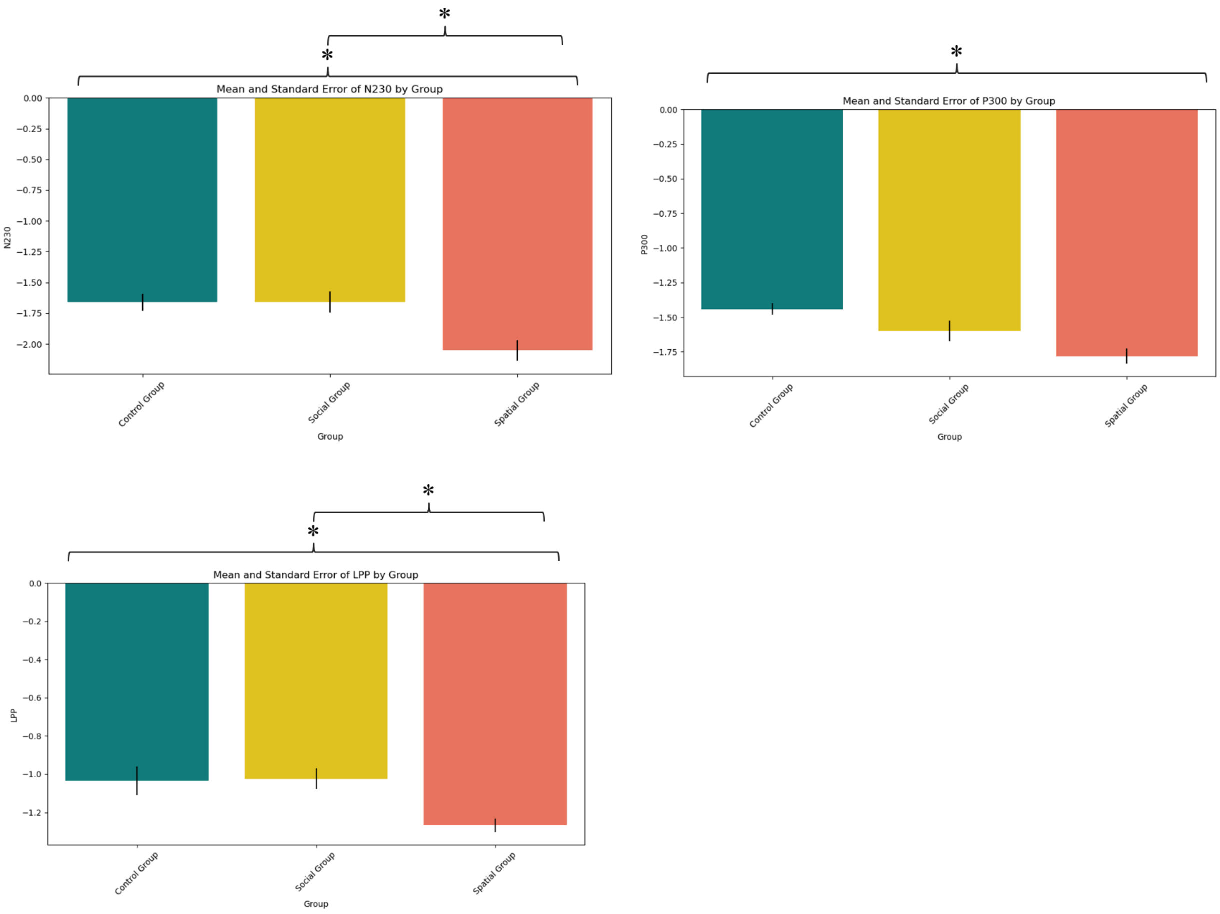

Incongruent Task

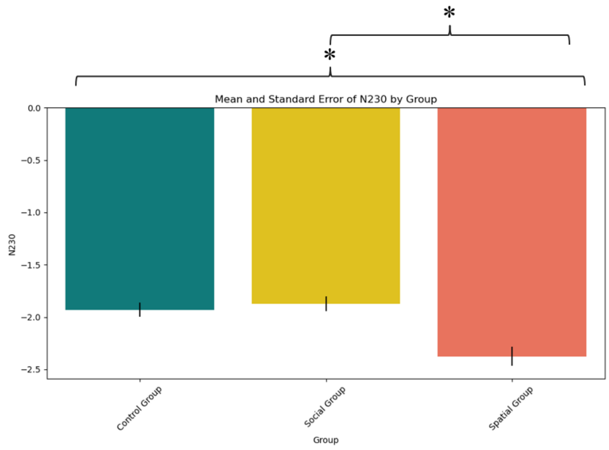

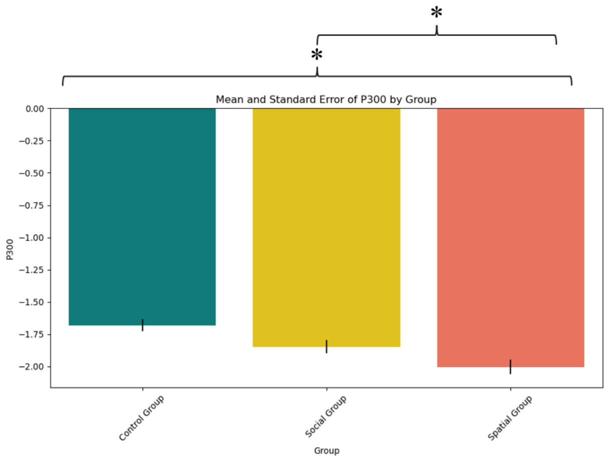

Table S11b Post Hoc Dunn's test for Social Task Congruent/Incongruent: Central electrodes

Variable	Group 1	Group 2	Dunn's (z)	Cohen's d	p-value
Congruent Task					
LPP	Control Group	Social Group	2.24	0.88	0.02
Incongruent Task					
P100	Control Group	Spatial Group	2,24	0.87	0.02
N200	Control Group	Spatial Group	2,09	0.72	0.04
LPP	Control Group	Social Group	2,01	0.77	0.04
LPP	Control Group	Spatial Group	2.18	0.86	0.03

Figure S6b Post Hoc Dunn's test for Social Task Congruent/Incongruent: Central Electrodes

Incongruent Task

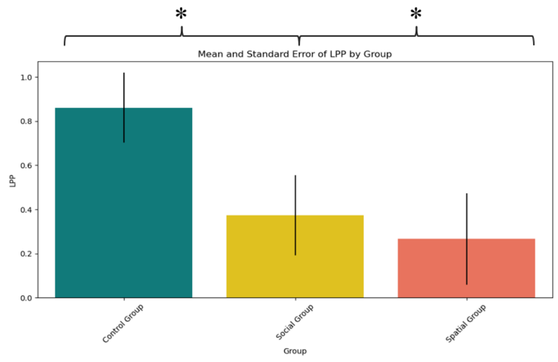

Table S11c Post Hoc Dunn's test for Social Task Congruent/Incongruent: Temporal electrodes

Variable	Group 1	Group 2	Dunn's (z)	Cohen's d	p-value
		Incongruent Task			
LPP	Control Group	Spatial Group	2.36	1.44	0.02

Figure S7c Post Hoc Dunn's test for Social Task Congruent/Incongruent: Temporal Electrodes

Incongruent Task

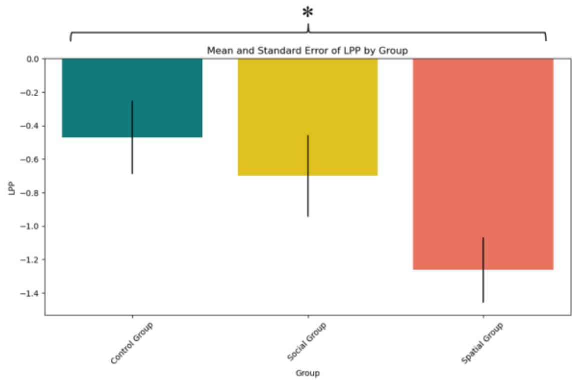

Table S11d Post Hoc Dunn's test for Social Task Congruent/Incongruent: Parietal Electrodes

Variable	Group 1	Group 2	Dunn's (z)	Cohen's d	p-value
		Congruent Task			
P300	Control Group	Social Group	-2.07	-0.78	0.04

P300	Control Group	Spatial Group	-3.66	-1.61	0.00
N400	Control Group	Social Group	-3.54	-1.54	0.00
N400	Control Group	Spatial Group	-3.44	-1.29	0.00
			Incongruent Task		
P300	Control Group	Spatial Group	-3.25	-1.26	0.00
N400	Control Group	Social Group	-3.14	-1.30	0.00
N400	Control Group	Social Group	-3.14	-1.30	0.00

Figure S6d Post Hoc Dunn's test for Social Task Congruent/Incongruent: Parietal Electrodes

Congruent Task

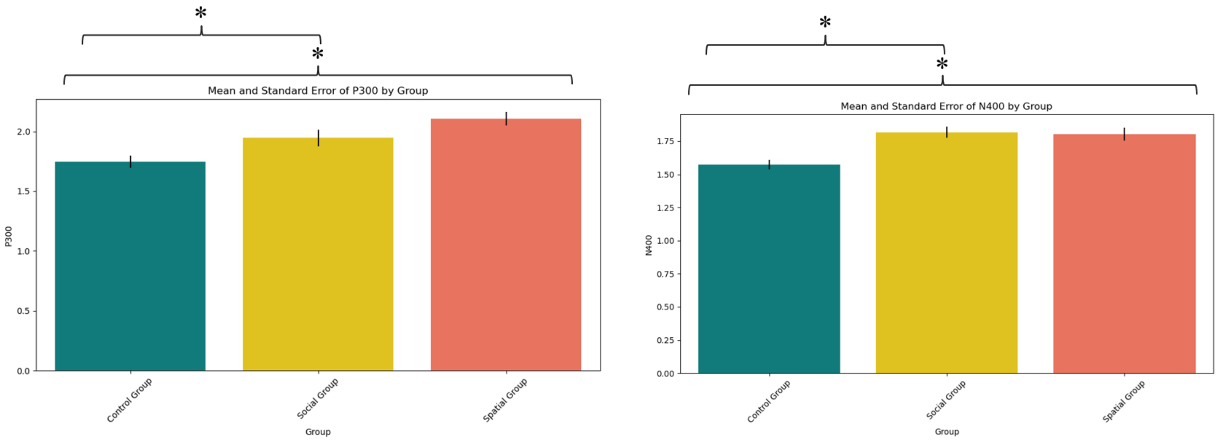

Incongruent Task

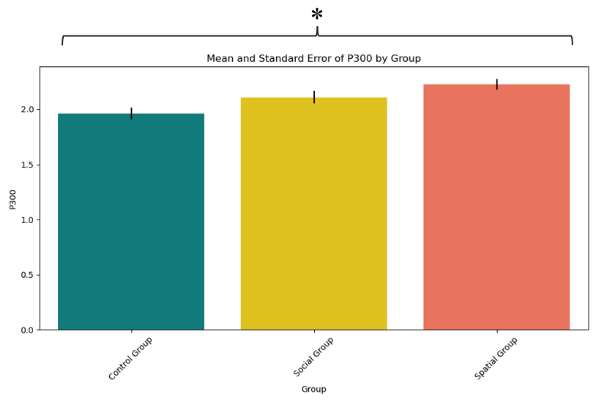

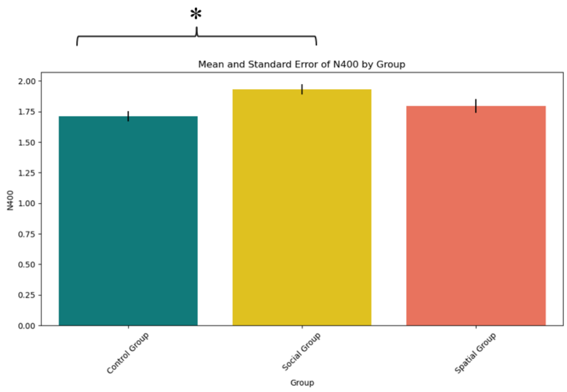

Description S1 Methods

The State-Trait Anxiety Inventory (STAI)

The State-Trait Anxiety Inventory (STAI), developed by Spielberger (1980), comprises two distinct subscales: the Trait Anxiety subscale (20 items) and the State Anxiety subscale (20 items). Participants are instructed for the Trait subscale to "Read each statement and then indicate on the scale how you generally feel." An illustrative item from this subscale is: "I worry too much over something that does not really matter." Responses are recorded on a Likert scale ranging from 1 ('rarely') to 4 ('almost always'), with the total score calculated as the sum of all item responses. Previous studies have established that the internal consistency of the Trait Anxiety subscale (Cronbach's α) ranges from .81 to .93 (Vitasari et al., 2011; Hallit et al., 2019). Conversely, the State Anxiety subscale assesses the individual's current state of anxiety by querying how respondents feel "right now." This subscale includes items designed to measure subjective experiences of apprehension, tension, nervousness, worry, and activation/arousal of the autonomic nervous system. Responses for this subscale are rated on a scale from 1 ('not at all') to 4 ('very much so'). The present study utilized the Russian adaptation of the STAI, which has demonstrated high internal consistency, with Cronbach's α values of .88 for the Trait Anxiety subscale and .89 for the State Anxiety subscale, in a large sample of adolescents (Likhanov et al., 2024).

Generalized Anxiety Disorder Questionnaire (GAD-7)

The Generalized Anxiety Disorder 7-item scale (GAD-7), developed by Spitzer et al. in 2006, is a self-report questionnaire designed to evaluate the presence of symptoms associated with generalized anxiety disorder (GAD). Participants are asked to reflect on their experiences over the past two weeks and respond to the prompt: "How often were you bothered by the following problems in the last two weeks?" Example items include statements such as "Feeling nervous, anxious, or on edge" and "Trouble relaxing." Responses are rated on a Likert scale ranging from 0 ("not at all") to 3 ("almost every day"), with the total score calculated as the sum of responses across all items. The GAD-7 has demonstrated strong psychometric properties, including high internal consistency (Cronbach's α = .85) and robust test-retest reliability (r = .83; Löwe et al., 2008). In our study, we utilized a Russian adaptation of the GAD-7, which also exhibited high internal consistency (Cronbach's α = .85) in a large sample of adolescents (Likhanov et al., 2024).

Appraisal of social concerns (ASC)

The questionnaire is specifically designed to assess social phobia and consists of 20 items that depict various social situations. Respondents are asked to indicate their level of concern regarding these situations on a scale from 0 to 100, where 0 represents "not at all concerned" and 100 indicates "extremely concerned." However, due to technical limitations that precluded the use of a slider scale, the current study employed a 5-point Likert scale, with response options ranging

from 1 ("not at all concerned") to 5 ("extremely concerned"). The test-retest reliability of the questionnaire has been established at r = .82, with an average time interval of 7.5 days between assessments (Telch et al., 2004). Additionally, a Russian adaptation of the questionnaire has demonstrated high internal consistency, with a Cronbach's alpha of .84 (Likhanov et al., 2024).

Spatial Anxiety Questionnaire (SA)

To evaluate spatial anxiety 10-item Way-Finding Strategy Scale was used (Lawton, 1994). Participants rated their anxiety levels in spatially demanding situations—such as navigation, way-finding, mental rotation, and spatial visualization—on a 5-point scale, where 1 indicated "not at all" and 5 represented "very much." An exploratory factor analysis revealed two primary factors within the scale: (A) Navigation Anxiety and (B) Rotation/Visualization Anxiety. The Navigation Anxiety factor included items such as "Finding your way around an intricate arrangement of streets," "Trying a new shortcut without using a map," and "Following somebody's instructions to get somewhere." This factor demonstrated excellent internal consistency, with a Cronbach's alpha of α = .86. The Rotation/Visualization Anxiety factor comprised items such as "Having to complete a complex jigsaw puzzle" and "Having to rotate objects in your mind." This factor also exhibited good internal consistency, with a Cronbach's alpha of α = .78 (Lawton, 1994; Malanchini et al., 2017).

Colour Stroop Tasks

The Classical Stroop Task was employed to assess cognitive problem solving towards neutral stimuli (Control Task). The task consisted of a series of color words (red, green, blue) presented in various ink colors that either matched or mismatched the semantic meaning of the words (Richards et al., 1992). For example, the word "green" might appear in red ink or in red ink.Overall there were 80 stimuli for the whole experiment. 40 of them were congruent and 40 of them were incongruent. Participants were instructed to press the keyboard buttons for the correct answer.

Social Stroop Tasks

The Social Stroop Task was utilized to evaluate cognitive processing in relation to emotion recognition within a social context. This task involved presenting participants with a series of facial expressions (specifically sad, happy, and angry) accompanied by written words "sad", "happy", and "angry" (Luciana et al., 2018) adapted by (Mohammed et al., 2022). In total, the experiment comprised 80 stimuli, with 40 stimuli categorized as congruent (where the facial expression and the written emotion aligned) and 40 as incongruent (where they did not align). Participants were instructed to respond by pressing the appropriate keys on the keyboard corresponding to the facial expression depicted. An example of the Emotional Stroop Task can be seen in Figure 1.

Spatial Flanker Task

The Spatial Flanker Task was employed to assess participants' attentional control and spatial problem solving. In this task, participants were presented with a series of trials where a central target stimulus was flanked by distractor stimuli on either side. The target stimulus consisted of an arrow pointing either left or right, while the distractors were arrows that could either point in the same direction as the target (congruent condition, 40 tasks) or in the opposite direction (incongruent condition, 40 tasks).
